# Prevalence and Genetic Characteristics of *Staphylococcus aureus* and *Staphylococcus argenteus* Isolates Harboring Panton-Valentine Leukocidin, Enterotoxins, and TSST-1 Genes from Food Handlers in Myanmar

**DOI:** 10.3390/toxins9080241

**Published:** 2017-08-04

**Authors:** Meiji Soe Aung, Thida San, Mya Mya Aye, San Mya, Win Win Maw, Khin Nyein Zan, Wut Hmone Win Htut, Mitsuyo Kawaguchiya, Noriko Urushibara, Nobumichi Kobayashi

**Affiliations:** 1 Department of Hygiene, School of Medicine, Sapporo Medical University, Sapporo 060-8556, Japan; kawaguchiya@sapmed.ac.jp (M.K.); noriko-u@sapmed.ac.jp (N.U.); nkobayas@sapmed.ac.jp (N.K.); 2 National Health Laboratory, Department of Health, Yangon 11191, Myanmar; drthidarsan@gmail.com (T.S.); dr.sanmya@gmail.com (S.M.); dr.khinnyeinzan@gmail.com (K.N.Z.); 3 University of Medical Technology, Mandalay 05072, Myanmar; prof.myamyaaye.umtm@gmail.com; 4 University of Medicine 2, Yangon 11031, Myanmar; winwinmawizumo@gmail.com; 5 New Yangon General Hospital, Yangon 11191, Myanmar; withmone.wh@gmail.com

**Keywords:** *Staphylococcus aureus*, *Staphylococcus argenteus*, food handler, Myanmar, Panton-Valentine leukocidin, enterotoxin, enterotoxin-like gene, TSST-1

## Abstract

Asymptomatic carriers of toxigenic *Staphylococcus aureus* are potential source of diseases, including food poisoning. Toxigenic potential and genetic traits of colonizing *S. aureus* were investigated for 563 healthy food handlers in Myanmar. Carriage of *S. aureus* was found in 110 individuals (19.5%), and a total of 144 *S. aureus* isolates were recovered from nasal cavities (110 isolates) and hands (34 isolates). Panton-Valentine leucocidin genes (*pvl*) were detected in 18 isolates (12.5%), among which 11 isolates were classified into *coa*-VIa, *agr* type III, and ST1930 (CC96) that had been also detected in *pvl*-positive clinical isolates in Myanmar. A *pvl*-positive, ST2250 nasal isolate was identified as *S. argenteus*, a novel coagulase-positive staphylococcus species. Toxic shock syndrome toxin-1 (TSST-1) gene was detected in five *pvl*-negative isolates. All of the 144 isolates harbored at least one of the 21 enterotoxin(-like) gene(s). The most prevalent enterotoxin(-like) gene was *selw* (98%), followed by *selx* (97%), *sei* (28%), *sely* (28%), *sem* (26%), *sel* (24%), and *sea* and *sec* (22% each). Considerable genetic diversity with five groups was detected for *selw*. The present study revealed the relatively high rate of *pvl*, as well as the wide distribution of enterotoxin(-like) genes among colonizing *S. aureus* in Myanmar.

## 1. Introduction

*Staphylococcus aureus* is one of the most common bacterial pathogens and causes various diseases, ranging from localized mild infections to invasive life-threatening diseases [1]. Particularly, methicillin-resistant *S. aureus* (MRSA), which acquired SCC*mec* in the genome, is recognized as the most leading cause of healthcare- as well as community-associated infections worldwide. Characteristic symptoms of infectious diseases due to this bacterium are known to be often associated with wide variety of virulence factors, including toxins, exoenzymes, and adhesins, which are secreted or linked to cell membrane. *S. aureus* strains producing Panton-Valentine leucocidin (PVL), a bicomponent cytotoxin encoded by prophage, show a propensity for skin and soft tissue infection (SSTI) and necrotizing pneumonia [2]. Staphylococcal enterotoxins (SEs) cause food poisoning with emesis [3], while toxic shock syndrome toxin-1 (TSST-1) is associated with severe multisystem illness [4]. SEs and TSST-1 are superantigens that have the ability to activate a large number of repertoires of T cells via binding to MHC class II and the Vβ chains of T cell receptor, leading to cytokine storm [5,6]. Various SEs and SE-like superantigens, including classical ones (SEA-SEE) and newly described toxins (SEG-SElY) have been reported [3,7]. 

It has been shown that approximately 30% of healthy individuals are asymptomatically colonized with *S. aureus*, including persistent and non-persistent carriers [8,9]. Colonization of *S. aureus* is considered to increase the risk of invasive diseases, including bacteremia in hospital settings [8,10]. For food poisoning outbreaks, *S. aureus* carriers in food handlers were described as the major source of contamination by epidemiological and molecular data [11,12]. Nasal carriage of *S. aureus*, particularly those producing enterotoxins, constitutes a main risk of contamination in food followed by possible food poisoning [13]. Therefore, the investigation of *S. aureus* carriage among food handlers and analysis of the prevalence of toxin genes in colonizing strains is important to prevent food contamination with toxigenic strains that may be related to food poisoning or other diseases.

To date, the prevalence of toxin genes among colonizing *S. aureus* in healthy individuals has been reported in many studies, revealing a generally high rate of SE genes, and a low prevalence of PVL gene (*pvl*, *lukS-PV-lukF-PV*) and TSST-1 gene (*tst-1*) [13,14,15,16]. In contrast, high rates of *pvl* (>10%) in colonizing *S. aureus* were shown by recent studies in African countries [17,18,19]. However, in Southeast Asia, little information is available on the prevalence of toxin genes in isolates from asymptomatic carriers. In Myanmar, a high rate (40%) of *pvl* among clinical isolates of *S. aureus* and their genetic traits was described [20], and the prevalence of SE genes was analyzed for limited number of isolates [21], but colonizing *S. aureus* has never been studied. In the present study, the prevalence of toxin genes was investigated for *S. aureus* strains isolated from healthy food handlers in Myanmar, and genotypes of isolates with toxin genes were analyzed. The prevalence of the newly identified SE-like genes, *selw*, *selx*, and *sely* [7,22,23], was also studied to add to the little information that exists on these toxin-like genes, and furthermore, *selw* was phylogenetically analyzed. The results indicated a universal prevalence of any SE(-like) genes, as well as a relatively high rate of *pvl* among colonizing *S. aureus* in food handlers.

## 2. Results

Among the 563 food handlers examined, 110 subjects (19.5%) were *S. aureus* carriers, and a total of 144 isolates (110 nasal and 34 hand isolates) were recovered. From 34 subjects, isolates were obtained from both sites. All the isolates were *mecA*-negative (i.e., methicillin-susceptible *S. aureus*; MSSA) and showed susceptibility to oxacillin, cefoxitin, and vancomycin. ACME-*arcA*, and exfoliative toxin genes were not detected in any isolate. *pvl* was detected in 18 isolates (15 nasal and three hand isolates) (12.5% in total, 13.6% and 8.8% in nasal and hand isolates, respectively), while *tst-1* was detected in five isolates (3.5%; four nasal and one hand isolates). All the 144 isolates harbored at least one of the 21 SE(-like) gene(s), among the 23 genes examined. The most prevalent enterotoxin gene was *selw* (98%), followed by *selx* (97%), *sei* (28%), *sely* (28%), *sem* (26%), *sel* (24%), *sea* and *sec* (22% each), and *seo* (21%). Three isolates were negative for *selw*, but solely harbored *sely*, or *sely* and *selx* (two and one isolate(s), respectively). Enterotoxin gene cluster (*egc*-1, *egc*-2), i.e., *seg-sei-sem-sen-seo*(*-selu*) was detected in 22 isolates (15%) (Table 1, Tables S1 and S2). 

All the isolates were classified into the 13 *coa* genotypes, with *coa*-VIIb being dominant (24 isolates), followed by VIa (22 isolates), and VIIa and Xa (18 isolates each). The incidence of *pvl* and SE(-like) genes differed depending on *coa* types. *pvl* was detected mostly in *coa*-VIa isolates, showing 55% (12/22) as *pvl*-positive rate. *sea* was detected most frequently in *coa*-VIa isolates (73%, 16/22), while *seb*, *sek*, *seq* were detected in *coa*-VIIb, *sec* and *sel* in both *coa*-VIa and Xa, and *egc*-2 in Ia, Va, and VIIb. In 17 food handlers, isolates from nasal cavity and hand showed the same *coa* type and toxin gene profiles, suggesting that these isolates are identical, although the genetic traits of isolates were different depending on the subject (Table S3). Only one food handler was a carrier of *pvl*-positive *coa*-VIa *S. aureus* in both nasal cavity and hand. In the remaining 17 subjects, isolates from the two sites showed different *coa* types or toxin gene profiles, indicating the colonization of different strains in nose and hand (Table S4). 

**Figure 1:**
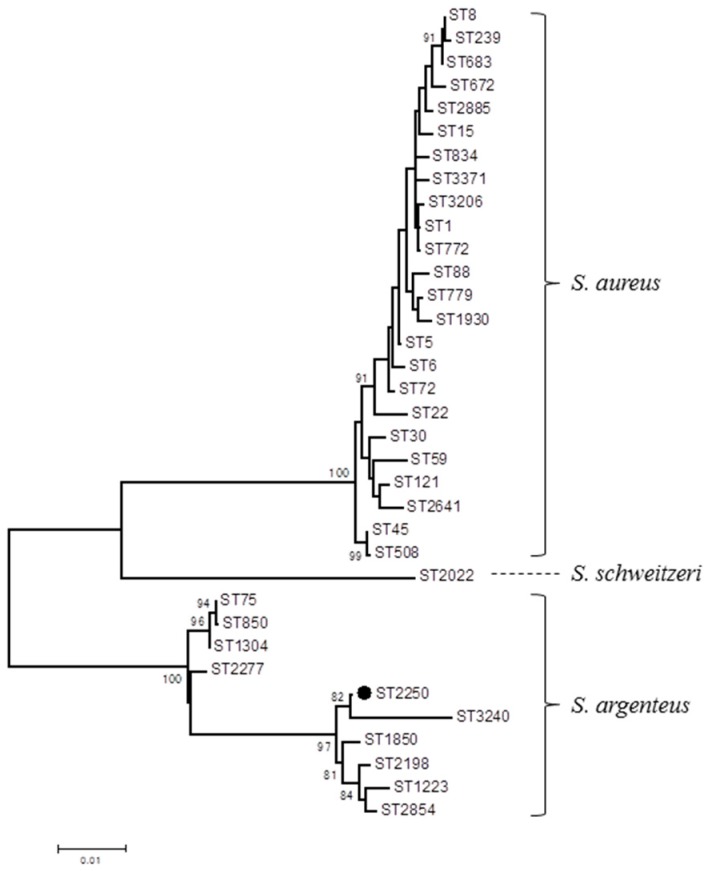
Phylogenetic dendrogram of *S. aureus* and two *S. aureus*-related species *S. argenteus* and *S. schweitzeri* based on the concatenated sequences of seven MLST loci using the maximum-likelihood method. This dendrogram contains *S. aureus* STs commonly found in Myanmar and other countries in Asia [20,21,27], representative of 10 *S. argenteus* STs and ST2022 of *S. schweitzeri* type strain FSA084. ST2250 identified in the present study is marked with a black circle. Bootstrap values >80% are shown. Scale bar, 0.01 substitutions per nucleotide.

STs and other genetic traits were analyzed for a total of 33 isolates, including all the 18 *pvl*-positive isolates, five *tst-1*-positive isolates, and other isolates with different *coa* types and toxin gene profiles (Table 2). *pvl*-positive isolates were classified into seven STs, among which ST1930 was dominant (61%; 11/18) and belonged to *coa*-VIa and *agr*-type III. PVL phage was mostly assigned to φPVL. Five *tst-1*-positive isolates belonged to ST5, ST8, ST508, or ST779, and *agr*-I, -II, or -III. *pvl*-positive isolates were susceptible to all of the seven antimicrobials examined, while *pvl*-negative, ST5, ST59, and ST672 isolates showed resistance to erythromycin and some other drugs. Two *coa*-XIb isolates (*pvl*-positive and -negative) were assigned to ST2250. These strains showed white colonies, representing a lack of carotenoid pigment. Nucleotide sequences of *nuc* gene and *orfX*-CRISPR (clustered regularly interspaced short palindromic repeat) element of ST2250 isolates showed 99.5% and 98% identities to those of *S. argenteus* strain MSHR1132 [24,25], respectively. In addition, the longer PCR amplicons of the nonribosomal peptide synthetase gene (NRPS), representing *S. argenteus* [26], were generated. Hence, these isolates were identified as *S. argenteus*, a novel coagulase-positive staphylococcus species closely related to *S. aureus*. Phylogenetic analysis of the concatenated sequences of seven MLST loci indicated that ST2250 detected in the present study clustered with ST1850 of *S. argenteus* type strain MSHR1132 within the *S. argenteus* lineage (Figure 1). The other three *coa*-XIb isolates detected in this study were also identified as *S. argenteus* because their *nuc* gene sequences were identical to that of the two ST2250 isolates. These five *S. argenteus* isolates were derived from the nares of five individuals (4.5% of 110 carriers).

**Figure 2:**
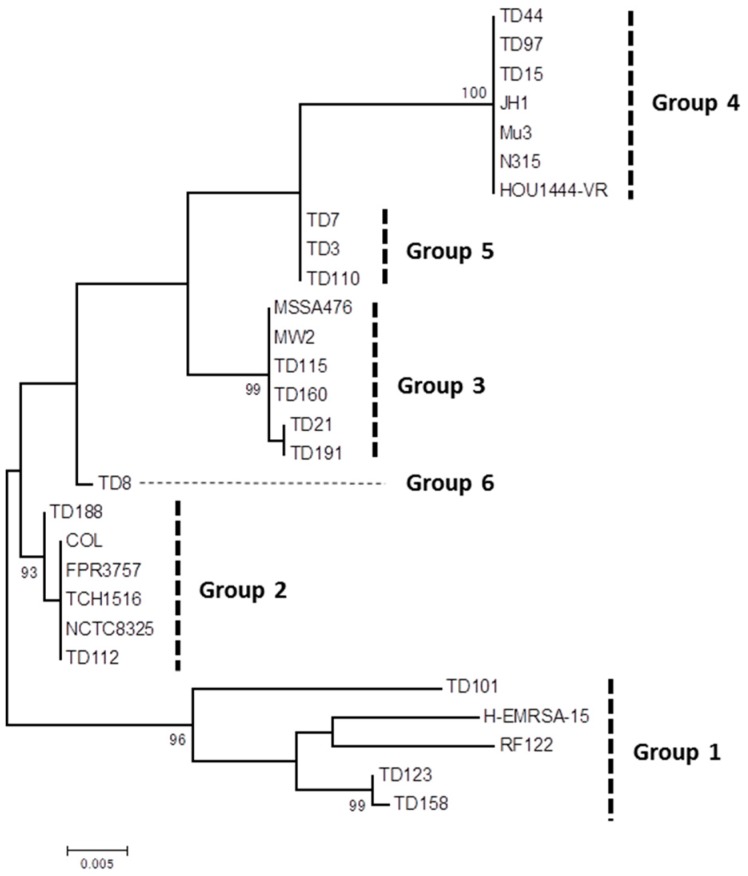
Phylogenetic dendrogram constructed from the nucleotide sequences of *selw* determined for 16 isolates in the present study, representative *S. aureus* strains reported previously [22]. Genetic groups assigned by this dendrogram are shown on the right. Bootstrap values >80% are shown. Scale bar, 0.005 substitutions per nucleotide.

One of the newly described SE-like genes, *selw*, was the most commonly detected gene among the isolates investigated in the present study. To analyze the genetic characteristics of this SE-like gene, whole open reading frame of *selw* was determined for 16 isolates with different genotypes and toxin gene profiles, and analyzed phylogenetically with previously reported *selw* sequences [22]. By the phylogenetic tree, six groups were discriminated (Figure 2). Nucleotide sequence identity was >99% within isolates with group 2–5, while it was lower within group 1 (Table S5). Among the different groups, sequence identity was 84–97%. Deduced SElW amino acid sequences of all the group 2 isolates and TD101 (group 1) were shorter than intact protein by 17 amino acids at the N-terminus (Figures S1 and S2). *selw* of group 4 isolates was considered to be a pseudogene encoding incomplete protein, as seen in strain N315 [22]. The amino acid sequences of all the SElW showed 31–43% identity and 68–78% similarity to that of SEA, and identical or similar amino acids between SElW and SEA were scattered throughout the protein sequence (Figure S3).

## 3. Discussion

Low prevalence of *pvl* (0–3%) in colonizing *S. aureus* from healthy community residents, healthcare workers, and children has been described in the US, Switzerland, China, and Korea [16,28,29,30,31], as well as those from hospitalized patients in Indonesia [32]. Slightly higher *pvl*-positive rates were shown among medical students in Saudi Arabia (6.3%) [33], healthy children in Greece (8.2%) [15], healthy individuals and patients in Indonesia (10.6%) [34], and healthcare workers in Madagascar (11.7%) [18]. In contrast, from tropical countries in the African continent (Burkina Faso, Congo, Gabon, Mali, Nigeria, Senegal), high detection rates (24–53%) of *pvl* were evidenced for *S. aureus* from nasal carriage [17,19,35,36,37,38]. Similarly, high *pvl*-positive rates were observed in local communities in south India, Ukraine, and Iowa state in the US [39,40,41]. In the present study, the detection rate of *pvl* among healthy food handlers (13.6%, nasal cavity) was found to be relatively higher than those from other Asian countries, Europe, and America, except for some local communities, suggesting that Myanmar is a prevalent area of *pvl*-carrying *S. aureus*, which may be relevant to the finding of a high *pvl*-positive rate (40%) among clinical isolates of *S. aureus* in Myanmar [20]. Furthermore, the major genotype among the *pvl*-positive isolates was ST1930 (CC96), which has not been reported for other studies on nasal isolates. Although ST1930 is a rare genotype, this ST was detected for *pvl*-positive *S. aureus* from hospitalized patients in Yangon, Myanmar [20], as well as in clinical isolates (both *pvl*-positive and negative) from two study sites in Bangladesh [42,43]. Accordingly, this type is estimated to be endemic in an area involving Myanmar and Bangladesh, and prevalent among healthy people, causing diseases occasionally. In addition, a *pvl*-positive ST88 isolate was also found in nasal carriage in the present study and was also reported as a cause of bacteremia in Myanmar [20]. Although our present study focused on workers in hotels/restaurants, colonizing *S. aureus* among food handlers working at street stalls in this country would be of significance to study, as their hygienic condition exposed to outside air appears to be worse than that in hotels and restaurants. 

The identification of the same genotype (ST, CC) of isolates from carriage and clinical specimens were shown in Gabon [35]. In addition, *pvl*-positive MRSA nasal colonizing isolates from the US children were assigned to ST8 [16], which represented the predominant community-acquired MRSA (USA300 clone) in the US [44]. Therefore, colonizing *S. aureus* is considered to be closely related to those causing infectious diseases. 

Previous studies on colonizing *S. aureus* isolates described the SE(-like) gene-positive rate as 68% (*sea*-*selj*) [13], 80% (*sea*-*selu*) among food handlers [45], and 81.7% (*sea*-*selj*) among healthcare workers [14]. However, our present study revealed that all of the colonizing isolates (i.e., 100%) harbored at least one of the 21 SE(-like) gene(s), through investigation for the presence of 23 SE(-like) genes including the recently described *selw*, *selx*, and *sely*, indicating a universal distribution of SE/superantigen genes in colonizing *S. aureus*. A remarkable finding was the high prevalence of *selw* (98%) and *selx* (97%). Similarly, in a study for blood and colonizing isolates in Austria, a high prevalence of *selx* (76.4%) was described, while the prevalence of *selw* was low (7.8%) [46]. Although we confirmed the presence of *selw* by sequencing in the present study, this SE-like gene was found to be genetically diverse and classified into six genetic groups. Among them, group 4 *selw* was considered a pseudogene, which probably encodes dysfunctional protein that may not act as SE/superantigen. In our study, SE(-like) genes were detected by multiplex PCR only, and the sequence of *selw* was determined for only 16 selected isolates. Thus, the prevalence of intact *selw* is not clear. To date, genetic diversity of SE genes has not yet been well characterized. Recently, genetic variants of major SE genes, *seb*, *sec*, and *sed* were described, with variation in *seb* promoter and truncated variant of *sed* [47]. It is possible that SE/superantigen activity may be different depending on variants of SEs. Therefore, for the evaluation of the toxigenicity of *S. aureus*, not only the distribution of SEs but also the genetic variants of SE genes should be considered. Mutated open reading frames were reported for alpha hemolysin, *selw*, and *selx* [46]. Because a high prevalence was also found for *selx* in our study, it would be of significance to analyze the diversity of *selx* as well as the prevalence of its potential pseudogene.

It was remarkable in the present study that *S. argenteus* (*pvl*-positive and -negative) was identified for the first time in Myanmar. *S. argenteus* is a novel species in the genus of *Staphylococcus*, and forms a part of *S. aureus* species complex with *S. schweitzeri* [24]. The type strain of *S. argenteus*, MSHR1132, a blood isolate in Australia, lacks pigment production genes, and has a *nucA* gene distinctive from *S. aureus*, as well as CRISPR element inserted into *orfX* [24,25]. After the first recognition of CC75 as *S. argenteus*, various STs have been reported to date, and this species has been isolated in New Zealand, Asia (Cambodia, Thailand, China), Europe (France, Belgium), Fiji, French Guiana, and Trinidad and Tobago [26,48,49,50]. A retrospective study in Thailand indicated that *S. argenteus* accounted for 4.1% of isolates originally identified as *S. aureus* [48]. In our present study, five isolates of *coa*-XIb (4.5% among apparent *S. aureus* carriers) were revealed to be *S. argenteus*, elucidating the distribution of *S. argenteus* among healthy individuals for the first time. ST2250, which was identified in Myanmar in this study, was also described in France, Belgium, and Thailand [48,49,50]. Although PVL genes were rarely detected [48], only two *S. argenteus* isolates in France belonging to ST2250 and ST2277 were revealed to harbor *pvl* [49]. The results from our present study indicated that *S. argenteus* is distributed in Southeast Asia, and *pvl*-positive ST2250, exhibiting the same genetic traits as reported in France, was colonizing in healthy individuals in Myanmar. Although *S. argenteus* is suggested to be less virulent than *S. aureus* [48], *pvl*-positive *S. aureus* may pose a concern for its increased virulence. Accordingly, further epidemiological surveillance is necessary to reveal the prevalence of *pvl* among this new species. 

## 4. Conclusions

A total of 144 colonizing *S. aureus* were recovered from 110 food handlers in Yangon, Myanmar. PVL genes were found in 18 isolates (12.5%), among which 11 isolates were classified into *coa*-VIa, *agr* type III, and ST1930 (CC96), that had been reported in Myanmar and Bangladesh. All of the 144 isolates harbored at least one of the SE(-like) gene(s) examined, with *selw* being dominant, while the TSST-1 gene was less prevalent. *selw* was revealed to have considerable diversity and classified into five groups. *S. argenteus*, a novel coagulase-positive *staphylococcus* species, was first identified in Myanmar for two ST2250, *pvl*-positive and -negative, nasal isolates. The present study revealed a relatively high prevalence of *pvl*, and a universal distribution of SE(-like) gene(s) among healthy food handlers in Myanmar. 

## 5. Materials and Methods

### 5.1. Study Subjects and Sample Collection, Bacterial Isolates

Study subjects were 563 food handlers (kitchen staff and wait staff) employed in 10 hotels (488 staff) and 22 restaurants (75 staff) in Yangon, Myanmar. Bacteriological examination of the food handlers was conducted as an occasional hygiene inspection on a voluntary basis by hotels/restaurants. During the study period from November 2012 to January 2014, food handlers from the hotels and restaurants were examined successively (generally two–four hotels/restaurants per month), and samples from each study subject were collected only once (in one–five days for a single hotel/restaurant) at the National Health Laboratory (NHL), Yangon, Myanmar. All of the study subjects were apparently in good health, having no skin infection on their hands such as abscess. From one food handler, nasal and hand swab samples were obtained using sterile cotton swabs containing physiological saline solution. The nasal swab was taken from the anterior nares (one swab for both nares), while the hand swab was taken from the wrist area of both hands. Primary isolation was conducted in the Bacteriology Section, NHL, Yangon, Myanmar. Bacterial isolates grown on mannitol salt agar plates and typical colonies were examined by conventional microbiological methods. Individual bacterial strains were stored in Microbank (Pro-Lab Diagnostics, Richmond Hill, ON, Canada) at −80 °C, and recovered when they were analyzed. The presence of staphylococcal 16 s rRNA, *nuc*, *mecA*, PVLgene (*lukS-PV*/*lukF-PV*) and ACME-*arcA* (arginine deiminase gene) was examined for all of the isolates by multiplex PCR assay, as described by Zhang et al. [51]. This study was approved and carried out according to the guidelines of the Research Ethics Committee of University of Medicine (2), Yangon, Myanmar.

### 5.2. Antimicrobial Susceptibility Testing

An antimicrobial susceptibility test was performed by agar disk diffusion method (Kirby-Bauer) using Mueller-Hinton agar for all of the *S. aureus* isolates. The bacterial suspensions with visually equivalent turbidity to 0.5 McFarland standards were prepared. The swab stick was dipped into the bacterial suspension, then taken out and squeezed on the wall of the test tube to discard extra suspension. The surface of the agar was uniformly inoculated using this swab. Seven antibiotic disks were placed on each plate. The plates were incubated at 35 °C overnight. Zones of inhibition on the agar plates were measured and the results were recorded and graded as resistance (R) and sensitive (S) according to the Clinical and Laboratory Standards Institute (CLSI 2013) standards. The antimicrobial agents used for susceptibility testing were oxacillin (1 μg), cefoxitin (30 μg), gentamicin (10 μg), levofloxacin (5 μg), clindamycin (2 μg), erythromycin (15 μg), and vancomycin (30 μg) disks (Oxoid, UK). *S. aureus* ATCC 25293 was used as a quality control standard.

### 5.3. Genotyping of *S. aureus*

For all of the *S. aureus* isolates, staphylocoagulase genotype was determined by multiplex PCR assay as previously described [52]. For the selected isolates, the accessory gene regulator (*agr*) group was determined by the PCR with specific primers [53]. The sequence type (ST) was determined according to the scheme of multilocus sequencing typing (MLST) [54], and the obtained ST data were further analyzed by eBURST to determine their clonal complex. PVL phage was typed by PCR assay as previously described [55,56].

### 5.4. Detection of SE(-Like)/TSST-1/Exfoliative Toxin Genes, Analysis of Selw

For each isolate, the presence of 23 SE(-like) genes (*sea-see*, *seg-selu*, *selw*, *selx*, *sely*), TSST-1 gene (*tst-1*), and exfoliative toxin genes (*eta*, *etb*, *etd*) was examined by multiplex/uniplex PCRs by using primers and PCR conditions as previously described [7,23,57]. For the detection of *selw*, primers selw-F2 (5′-GGAATTTTTACAAATTCAGCG-3′) and selw-R2 (5′-CTACATTGCGTTTTATTGGTTG-3′) were used for PCR (product size; 323 bp). The nucleotide sequence of *selw* was determined directly from PCR product with primers COL-SELW1 (5′-TTATGTCTCGCTCTCAAATG-3′) and COL-SELW2 (5′-CTGCTGTTTTTATTTGTAAC-3′), using the BigDye Terminator v3.1 Cycle Sequencing kit (Applied Biosystems, Foster City, CA, USA) on an automated DNA sequencer (ABI PRISM 3100). Phylogenetic tree was constructed by the maximum likelihood method using MEGA software [58] (Version 6.0). The tree was statistically supported by bootstrapping with 1000 replicates. Clustal W program was used for multiple alignments of nucleotide and amino acid sequences of *selw* and *sea*. The pairwise alignment of nucleotide and amino acid sequences and the calculation of identity were performed by LALIGN server. Nucleotide sequences of *selw* of 16 *S. aureus* isolates were deposited in the GenBank database under accession numbers KX655702–KX655717.

### 5.5. Sequence Analysis of *S. argenteus*

Nuclease gene and *orfX*-*cas1* sequences of an *S. argenteus* isolate TD162 and TD171 were determined by PCR and direct sequencing, and the sequence data of TD162 were deposited in the GenBank database under accession numbers MF167422 and MF167423, respectively. Primers used for PCR amplification of these genes are listed in Table S6. PCR to amplify the nonribosomal peptide synthetase (NRPS) gene to discriminate *S. argenteus*/*S. schweitzeri* from *S. aureus* was performed as previously described [26]. The phylogenetic tree of concatenated sequences of seven MLST loci of *S. argenteus* detected in the present study and those reported previously [48], STs of *S. aureus* reported in Asia including Myanmar [20,21,27], and an ST of *S. schweitzeri* [24] was constructed by the maximum likelihood method using MEGA6 software. 

## Figures and Tables

**Table 1:** Detection of PVL, TSST-1, and enterotoxin(-like) genes in nasal and hand isolates.

Toxin Genes	*coa*-Genotype (No. of Isolates)													Total *n* = 144 (%)
	Ia (7)	IIa (3)	IIIa (15)	IVb (11)	Va (10)	Vb (9)	VIa (22)	VIc (1)	VIIa (18)	VIIb (24)	VIIIa (1)	Xa (18)	XIb (5)	
*lukS-PV-lukF-PV* (PVL)	0	0	2	0	1	0	12	0	2	0	0	0	1	18 (12.5%)
*tst-1* (TSST-1)	0	2	2	0	0	0	0	0	0	1	0	0	0	5 (3.5%)
Enterotoxin (-like) genes *^1^														
*sea*	0	0	0	9	1	0	16	0	2	3	0	0	0	31 (22%)
*seb*	0	1	5	0	2	0	1	0	0	10	0	1	0	20 (14%)
*sec*	0	1	0	0	1	2	13	0	1	4	0	9	0	31 (22%)
*seg*	6	1	0	0	5	0	3	0	0	9	1	1	0	26 (18%)
*seh*	0	0	0	0	0	0	0	0	2	1	0	2	0	5 (3%)
*sei*	6	2	0	0	8	0	8	0	1	11	1	4	0	41 (28%)
*selj*	0	0	0	0	0	0	0	0	0	0	0	5	0	5 (3%)
*sek*	0	0	3	1	1	0	0	0	4	11	0	0	0	20 (14%)
*sel*	1	0	1	0	0	4	10	0	2	6	1	9	0	34 (24%)
*sem*	6	2	1	0	9	0	4	0	0	10	1	4	0	37 (26%)
*sen*	6	1	0	0	8	0	1	0	0	10	0	2	0	28 (19%)
*seo*	6	2	0	0	8	0	3	0	0	9	1	1	0	30 (21%)
*sep*	0	0	7	0	1	0	0	0	0	5	0	0	0	13 (9%)
*seq*	0	0	3	1	1	0	0	0	3	8	0	0	0	16 (11%)
*ser*	0	0	0	0	0	0	0	0	0	0	0	4	0	4 (3%)
*ses*	0	0	0	0	0	0	0	0	0	0	0	4	0	4 (3%)
*set*	0	0	0	0	0	0	0	0	0	0	0	4	0	4 (3%)
*selu*	6	1	0	0	5	0	3	0	0	6	1	1	0	23 (16%)
*selw*	7	3	15	11	10	9	22	1	18	24	1	18	2	141 (98%)
*selx*	7	3	15	11	10	9	22	1	18	23	1	18	1	139 (97%)
*sely*	2	0	1	1	0	0	1	0	11	11	0	9	4	40 (28%)
Enterotoxin genes clusters *^2^/combinations (initials of genes)														
*g*, *i*, *m*, *n*, *o*, *u*	6	1	1	0	4	0	1	0	0	5	0	1	0	19 (13%)
*g*, *i*, *m*, *n*, *o*	0	0	0	0	0	0	0	0	0	3	0	0	0	3 (2%)
*j*, *r*, *s*, *t*	0	0	0	0	0	0	0	0	0	0	0	4	0	4 (3%)
*k*, *q*	0	0	3	1	1	0	0	0	3	8	0	0	0	16 (11%)

*^1^ *sed* and *see* were not detected in any strain; *^2^ *g-i-m-n-o*, *egc*-1; *g-i-m-n-o-u*, *egc*-2.

**Table 2:** Genotypes, PVL/TSST-1/enterotoxin genes and drug resistance of 33 *S. aureus* isolates analyzed (18 PVL-positive isolates and 15 PVL-negative isolates, including two *S. argenteus* isolates).

Strain ID ^a^	Age/Sex	Specimen	*coa* Type	ST	CC	*agr* Type	PVL Phage	Enterotoxin (-Like)/TSST-1 Genes ^b^	Drug Resistance ^c^
TD8	32/M	Nasal swab	IIIa	ST88	CC88	III	ΦPVL	*selx*, *selw*	
TD188	32/F	Nasal swab	IIIa	ST683	CC8	I	ΦPVL	*seg*, *sei*, *sem*, *sen*, *seo*, *seu*, *sep*, *selx*, *selw*	
TD158	45/F	Hand swab	Va	ST45	CC45	IV	ΦPVL	*sea*, *seg*, *sei*, *sem*, *sen*, *seo*, *seu*, *selx*, *selw*	
TD1	28/M	Nasal swab	VIa	ST1930	CC96	III	ΦPVL	*sea*, *sec*, *sei*, *sel*, *selx*, *selw*	
TD3	25/F	Nasal swab	VIa	ST1930	CC96	III	ΦSa2mw	*sea*, *sec*, *sei*, *selx*, *selw*	
TD7	29/M	Nasal swab	VIa	ST1930	CC96	III	ΦPVL	*sea*, *sec*, *selx*, *sely*, *selw*	
TD19	22/M	Nasal swab	VIa	ST1930	CC96	III	ΦPVL	*sea*, *sec*, *sei*, *sel*, *selx*, *selw*	
TD25	18/M	Nasal swab	VIa	ST1930	CC96	III	ΦPVL	*sea*, *sec*, *sel*, *selx*, *selw*	
TD31	27/M	Nasal swab	VIa	ST1930	CC96	III	ΦPVL	*sea*, *sec*, *sel*, *selx*, *selw*	
TD47	25/M	Nasal swab	VIa	ST1930	CC96	III	ΦPVL	*sea*, *sec*, *sei*, *sem*, *selx*, *selw*	
TD110	28/M	Nasal swab	VIa	ST1930	CC96	III	ΦPVL	*sea*, *sec*, *sel*, *selx*, *selw*	
TD157	28/M	Hand swab	VIa	ST1930	CC96	III	ΦPVL	*sea*, *sec*, *sel*, *selx*, *selw*	
TD149	24/M	Nasal swab	VIa	ST3206	CC1	II	ΦPVL	*sea*, *seg*, *sei*, *sem*, *seo*, *seu*, *selx*, *selw*	
TD161	23/M	Nasal swab	VIa	ST1930	CC96	III	ΦPVL	*sea*, *sec*, *sel*, *selx*, *selw*	
TD170	29/F	Nasal swab	VIa	ST1930	CC96	III	ΦPVL	*sea*, *sec*, *sel*, *selx*, *selw*	
TD115	24/F	Nasal swab	VIIa	ST1	CC1	III	ΦPVL	*seh*, *sek*, *selx*, *selw*	
TD160	24/F	Hand swab	VIIa	ST1	CC1	III	ΦPVL	*sek*, *seq*, *selx*, *selw*	
TD162	23/F	Nasal swab	XIb	ST2250 ^d^	CC2250	NT ^e^	ΦPVL	*sely*	
TD97	27/M	Hand swab	Ia	ST672	CC672	I	(-)	*seg*, *sei*, *sem*, *sen*, *seo*, *seu*, *selx*, *sely*, *selw*	LVX, ERY
TD15	27/M	Nasal swab	IVb	ST6	CC6	I	(-)	*sea*, *selx*, *selw*	
TD40	27/M	Nasal swab	Va	ST2641		II	(-)	*sei*, *sem*, *sen*, *seo*, *selx*, *sew*	
TD101	32/M	Nasal swab	VIIb	ST2885		I	(-)	*seb*, *sek*, *seq*, *selx*, *sely*, *selw*	GEN, LVX, CLI, ERY
TD104	29/M	Hand swab	VIIb	ST59	CC59	I	(-)	*seb*, *sek*, *sep*, *seq*, *selx*, *sely*, *selw*	GEN, LVX, CLI, ERY
TD21	25/F	Nasal swab	Xa	ST15	CC15	II	(-)	*sej*, *ses*, *set*, *ser*, *selx*, *selw*	
TD44	28/M	Nasal swab	IIa	ST5	CC5	II	(-)	*seg*, *sei*, *sem*, *sen*, *seo*, *seu*, *selx*, *selw*, *tst-1*	CLI, ERY
TD191	31/M	Nasal swab	IIa	ST779	CC779	III	(-)	*seb*, *selx*, *selw*, *tst-1*	ERY
TD112	23/F	Nasal swab	IIIa	ST8	CC8	I	(-)	*sek*, *sel*, *seq*, *selx*, *selw*, *tst-1*	
TD159	23/F	Hand swab	IIIa	ST8	CC8	I	(-)	*sek*, *sem*, *seq*, *selx*, *selw*, *tst-1*	
TD123	25/M	Nasal swab	VIIb	ST508	CC45	I	(-)	*sec*, *seg*, *sei*, *sel*, *sem*, *sen*, *seu*, *selx*, *selw*, *tst-1*	
TD20	22/M	Hand swab	VIa	ST1930	CC96	III	(-)	*sea*, *sec*, *sei*, *sel*, *selx*, *selw*	
TD99	25/F	Nasal swab	VIa	ST3371		I	(-)	*selx*, *selw*	
TD153	31/M	Nasal swab	VIa	ST3206	CC1	II	(-)	*seg*, *sei*, *sem*, *sen*, *seo*, *seu*, *selx*, *selw*	
TD171	25/M	Nasal swab	XIb	ST2250 ^d^		NT ^e^	(-)	*sely*	

^a^ Three pairs of nasal and hand isolates (TD110 and TD157, TD115 and TD160, and TD112 and TD159) were derived from the same individuals; ^b^ The following genes were not detected in any strain : *sed*, *see*, *eta*, *etb*, *etd*; ^c^ CLI, Clindamycin; ERY, Erythromycin; GEN, Gentamicin; LVX, Levofloxacin. None of the strains showed resistance to oxacillin, cefoxitin and vancomycin; ^d^ *S. argenteus*; ^e^ Non typable.

## Supplementary Materials

The following are available online at www.mdpi.com/2072-6651/9/8/241/s1, Figure S1: Alignment of nucleotide sequences of intact selw genes and pseudogenes encoding truncated SElW; Figure S2: Alignment of amino acid sequences of intact SElW (a), truncated SElW with an intact SElW of strain COL (b). Amino acid numbers are indicated for intact SElW; Figure S3: Alignment of amino acid sequences of intact SElW and SEA, Table S1: Detection of PVL, TSST-1, and enterotoxin(-like) genes in nasal isolates, Table S2: Detection of PVL, TSST-1, and enterotoxin(-like) genes in hand isolates, Table S3: Identical *coa* types and toxin gene profile in same individual, Table S4: Different *coa* types and/or toxin gene profile in same individual, Table S5: Identities (Percentage) of *selw* nucleotide sequences (upper right) and deduced amino acid sequences (lower left) among the selected strains, Table S6: Primers used for PCR amplification of *S. argenteus* genes.
