# Supplementary material for: Prevalence and Genetic Characteristics of Staphylococcus aureus and Staphylococcus argenteus Isolates Harboring Panton-Valentine Leukocidin, Enterotoxins, and TSST-1 Genes from Food Handlers in Myanmar
Source: Toxins (Basel). 2017 Aug 4;9(8):241. doi: 10.3390/toxins9080241 (PMC5577575; doi:10.3390/toxins9080241)
Supplement: Supplementary file 1 [file toxins-09-00241-s001.pdf]

Supplementary Materials: Prevalence and Genetic Characteristics of *Staphylococcus aureus* and *Staphylococcus argenteus* Isolates Harboring Panton-Valentine Leukocidin, Enterotoxins, and TSST-1 Genes from Food Handlers in Myanmar

Meiji Soe Aung, Thida San, Mya Mya Aye, San Mya, Win Win Maw, Khin Nyein Zan, Wut Hmone Win Htut, Mitsuyo Kawaguchiya, Noriko Urushibara and Nobumichi Kobayashi

```
                                      30                            60
COL       TCAAATGTAAATTAATAATTGTTTTGGGGGAGTTTGAAGTTAAATATTTAACAGGATTTA
TD112     TCAAATGTAAATTAATAATTGTTTTGGGGGAGTTTGAAGTTAAATATTTAACAGGATTTA
TD188     TCAAATGTAAATTAATAATTGTTTTGGGGGAGTTTGAAGTTAAATATTTAACAGGATTTA
TD8       TCAAATGTAAATTAATAATTGTTTTGGGGGAGTTTGAAGTTAAATATTTAACAGGATTTA
TD3       TCAAATGTAAATTAATAATTGTTTTGGGGGAGTTTGAAGTTAAATATTTAACAGGATTTA
TD110     TCAAATGTAAATTAATAATTGTTTTGGGGGAGTTTGAAGTTAAATATTTAACAGGATTTA
TD7       TCAAATGTAAATTAATAATTGTTTTGGGGGAGTTTGAAGTTAAATATTTAACAGGATTTA
N315      TCAAATGTAAATTAATAATTGTTTTGGGGGAGTTTGAAGTTAAATATTTAACAGGATTTA
TD15      TCAAATGTAAATTAATAATTGTTTTGGGGGAGTTTGAAGTTAAATATTTAACAGGATTTA
TD44      TCAAATGTAAATTAATAATTGTTTTGGGGGAGTTTGAAGTTAAATATTTAACAGGATTTA
TD97      TCAAATGTAAATTAATAATTGTTTTGGGGGAGTTTGAAGTTAAATATTTAACAGGATTTA
TD191     TCAAATGTAAATTAATAATTGTTTTGGGG-AGTTTGAAGTTAAATATTTAACAGGATTTA
TD21      TCAAATGTAAATTAATAATTGTTTTGGGG-AGTTTGAAGTTAAATATTTAACAGGATTTA
TD160     TCAAATGTAAATTAATAATTGTTTTGGGG-AGTTTGAAGTTAAATATTTAACAGGATTTA
TD115     TCAAATGTAAATTAATAATTGTTTTGGGG-AGTTTGAAGTTAAATATTTAACAGGATTTA
TD123     TCAAATGTAAATTAATAATTGTTTTGGGGGAGTTTGAAGTTAAATATTTAACAGGATTTA
TD158     TCAAATGTAAATTAATAATTGTTTTGGGGGAGCTTGAAGTTAAATATTTAACAGGATTTA
TD101     TCAAATGTAAATTAATAATTGTTTTGGGG-AGTTTGAAGTTAAATATTTAACAGGATTTA
          ***************************** ** ***************************

                                                                   120
COL       TTTTAATATTATTGTTAGAAGGAATTTTTACAAATTCAGCGAGTGCAATCGAATATTCAG
TD112     TTTTAATATTATTGTTAGAAGGAATTTTTACAAATTCAGCGAGTGCAATCGAATATTCAG
TD188     TTTTAATATTATTGTTAGAAGGAATTTTTACAAATTCAGCGAGTGCAATCGAATATTCAG
TD8       TTTTAATATTATTGTTAGAAGGAATTTTTACAAATTCAGCGAGTGCAATCGAATATTCAG
TD3       TTTTAATATTATTGTTAGAAGGAATTTTTACAAATTCAGCGAGTGCAATCGAATATTCAG
TD110     TTTTAATATTATTGTTAGAAGGAATTTTTACAAATTCAGCGAGTGCAATCGAATATTCAG
TD7       TTTTAATATTATTGTTAGAAGGAATTTTTACAAATTCAGCGAGTGCAATCGAATATTCAG
N315      TTTTAATATTATTGTTAGAAGGAATTTTTACAAATTCAGCGAGTGCAATCGAATATTCAG
TD15      TTTTAATATTATTGTTAGAAGGAATTTTTACAAATTCAGCGAGTGCAATCGAATATTCAG
TD44      TTTTAATATTATTGTTAGAAGGAATTTTTACAAATTCAGCGAGTGCAATCGAATATTCAG
TD97      TTTTAATATTATTGTTAGAAGGAATTTTTACAAATTCAGCGAGTGCAATCGAATATTCAG
TD191     TTTTAATATTATTGTTAGAAGAAATTTTGACAAATTCAGCAAGTGCAATCGAATATTCAG
TD21      TTTTAATATTATTGTTAGAAGAAATTTTGACAAATTCAGCAAGTGCAATCGAATATTCAG
TD160     TTTTAATATTATTGTTAGAAGAAATTTTGACAAATTCAGCAAGTGCAATCGAATATTCAG
TD115     TTTTAATATTATTGTTAGAAGAAATTTTGACAAATTCAGCAAGTGCAATCGAATATTCAG
TD123     TTTTAATATTATTGTTAGAAGGAATTTTTACAAATTCAGCAAGTGCAATCGAATATTCAG
TD158     TTTTAATATTATTGTTAGAAGGAATTTTTACAAATTCAGCAAGTGCAATCGAATATTCAG
TD101     TTTTAATATTATTGTTAGAAGAAATTTTGACAAATTCAGTAAGTGCAATCGAATATTCAG
          ********************* ****** **********  *******************
```

```
                                                                         180
COL           ACTTACATCATAAAAGTAAGTTTGATTCAAAGCGTCTAAGTAATGCTAAGATGTCATTCA
TD112         ACTTACATCATAAAAGTAAGTTTGATTCAAAGCGTCTAAGTAATGCTAAGATGTCATTCA
TD188         ACTTACATCATAAAAGTAAGTTTGATTCAAAGCGTCTAAGTAATGCTAAGATGTCATTCA
TD8           ACTTACATCATAAAAGTAAGTTTGATTCAAAGCGTCTAAGTAATGCTAAGATGTCATTCA
TD3           ACTTACATCATAAAAGTAAGTTTGATTCAAAGCGTCTAAGTAATGCTAAGATGTCATTCA
TD110         ACTTACATCATAAAAGTAAGTTTGATTCAAAGCGTCTAAGTAATGCTAAGATGTCATTCA
TD7           ACTTACATCATAAAAGTAAGTTTGATTCAAAGCGTCTAAGTAATGCTAAGATGTCATTCA
N315          ACTTACATCATAAAAGTAAGTTTGATTCAAAGCGTCTAAGTAATGCTAAGATGTCATTCA
TD15          ACTTACATCATAAAAGTAAGTTTGATTCAAAGCGTCTAAGTAATGCTAAGATGTCATTCA
TD44          ACTTACATCATAAAAGTAAGTTTGATTCAAAGCGTCTAAGTAATGCTAAGATGTCATTCA
TD97          ACTTACATCATAAAAGTAAGTTTGATTCAAAGCGTCTAAGTAATGCTAAGATGTCATTCA
TD191         ACTTACATCATAAAAGTAAGTTTGATTCAAAGCGTCTAAGTAATGCTAAGATGTCATTCA
TD21          ACTTACATCATAAAAGTAAGTTTGATTCAAAGCGTCTAAGTAATGCTAAGATGTCATTCA
TD160         ACTTACATCATAAAAGTAAGTTTGATTCAAAGCGTCTAAGTAATGCTAAGATGTCATTCA
TD115         ACTTACATCATAAAAGTAAGTTTGATTCAAAGCGTCTAAGTAATGCTAAGATGTCATTCA
TD123         ACTTACATCATAAAAGTAAACTTGATTCAAAGCGTCTATATAATGCTAAGGTGTCATTTG
TD158         ACTTACATCATAAAAGTAAACTTGATTCAAAGCGTCTATATAATGCTAAGGTGTCATTTG
TD101         ACTTACATCATAAAAGTAAACTAAATTCAAAGCGTCTATATAATGCTAAGGTGTCATTTG
              *******************  *  **************  ********** *******

                                                                         240
COL           TCAATCCAACTCAGCTTGAAAATAAAAACACAAACGATAGACTGTTGAAGCATGATTTGT
TD112         TCAATCCAACTCAGCTTGAAAATAAAAACACAAACGATAGACTGTTGAAGCATGATTTGT
TD188         TCAATCCAACTCAGCTTGAAAATAAAAACACAAACGATAGACTGTTGAAGCATGATTTGT
TD8           TCAATCCAACTCAGCTTGAAAATAAAAACACAAAAGATAGACTGTTGAAGCATGATTTGT
TD3           TCAATCCAACTCAGCTTGAAAATAAAAACACAAACGATAGACTGTTGAAGCATGATTTGT
TD110         TCAATCCAACTCAGCTTGAAAATAAAAACACAAACGATAGACTGTTGAAGCATGATTTGT
TD7           TCAATCCAACTCAGCTTGAAAATAAAAACACAAACGATAGACTGTTGAAGCATGATTTGT
N315          TCAATCCAACTCAGCTTGAAAATAAAAACACAAACGATAGACTGTTGAAGCATGATTTGT
TD15          TCAATCCAACTCAGCTTGAAAATAAAAACACAAACGATAGACTGTTGAAGCATGATTTGT
TD44          TCAATCCAACTCAGCTTGAAAATAAAAACACAAACGATAGACTGTTGAAGCATGATTTGT
TD97          TCAATCCAACTCAGCTTGAAAATAAAAACACAAACGATAGACTGTTGAAGCATGATTTGT
TD191         TCAATCCAACTCAGCTTGAAAATAAAAACACAAACGATAGACTGTTGAAGCATGATTTGT
TD21          TCAATCCAACTCAGCTTGAAAATAAAAACACAAACGATAGACTGTTGAAGCATGATTTGT
TD160         TCAATCCAACTCAGCTTGAAAATAAAAACACAAACGATAGACTGTTGAAGCATGATTTGT
TD115         TCAATCCAACTCAGCTTGAAAATAAAAACACAAACGATAGACTGTTGAAGCATGATTTGT
TD123         CTAATCCAACTGATCTTGAAAATAAAAAAACAAACGATAGACTGTTGAAGCATGATTTGT
TD158         CTAATCCAACTGATCTTGAAAATAAAAAAACAAACGATAGACTGTTGAAGCATGATTTGT
TD101         CTAATCCAACTGATCTTGAAAATAAAAACACAAACGATAGACTGTTGAAGCATGATTTGT
                ********* * ************** ***** *************************

                                                                         300
COL           TATTTCATGACATGTTCGTAAATGATGATTGGAAAAAGGATTTTAAAGTTGAATTTGAAA
TD112         TATTTCATGACATGTTCGTAAATGATGATTGGAAAAAGGATTTTAAAGTTGAATTTGAAA
TD188         TATTTCATGACATGTTCGTAAATGATGATTGGAAAAAGGATTTTAAAGTTGAATTTGAAA
TD8           TATTTCATGACATGTTCGTAAATGATGATTGGAAAAAGGATTTTAAAGTTGAATTTGAAA
TD3           TATTTCATGACATGTTCGTAAATGATGATTGGAAAAAGGATTTTAAAGTTGAATTCGAAA
TD110         TATTTCATGACATGTTCGTAAATGATGATTGGAAAAAGGATTTTAAAGTTGAATTCGAAA
TD7           TATTTCATGACATGTTCGTAAATGATGATTGGAAAAAGGATTTTAAAGTTGAATTCGAAA
N315          TATTTCATGACATGTTCGTAAATGTTGCTTCGAAAAAGGATTTTAAAGTTGAATTCGAAA
TD15          TATTTCATGACATGTTCGTAAATGTTGCTTCGAAAAAGGATTTTAAAGTTGAATTCGAAA
TD44          TATTTCATGACATGTTCGTAAATGTTGCTTCGAAAAAGGATTTTAAAGTTGAATTCGAAA
TD97          TATTTCATGACATGTTCGTAAATGTTGCTTCGAAAAAGGATTTTAAAGTTGAATTCGAAA
TD191         TATTTCATGACATGTTCGTAAATGATGATTGGAAAAAAGATTTTAAAGTTGAATTCGAAA
TD21          TATTTCATGACATGTTCGTAAATGATGATTGGAAAAAAGATTTTAAAGTTGAATTCGAAA
TD160         TATTTCATGACATGTTCGTAAATGATGATTGGAAAAAAGATTTTAAAGTTGAATTCGAAA
TD115         TATTTCATGACATGTTCGTAAATGATGATTGGAAAAAAGATTTTAAAGTTGAATTCGAAA
TD123         TATTTCATGACATGTTCGTAAATGATGATTGGAAAAAAGATTTTAAAGTTGAATTCGAAA
TD158         TATTTCATGACATGTTCGTAAATGATGATTGGAAAAAAGATTTTAAAGTTGAATTCGAAA
TD101         TATTTCATGACATGTTCGTAAATGTTGCTTCGAAAAAGGATTTTAAAGTTGAATTCGAAA
              ************************ ** ** ****** ***************** ****
```

```
                                                                         360
COL       ATGAGGCACTTTCAAAGAAATTTATAAATAAGGATATCGATATATTTGCTGGAAATTATG
TD112     ATGAGGCACTTTCAAAGAAATTTATAAATAAGGATATCGATATATTTGCTGGAAATTATG
TD188     ATGAGGCACTTTCAAAGAAATTTATAAATAAGGATATCGATATATTTGCTGGAAATTATG
TD8       ATGAGGCACTTTCAAAGAAATTTATAAATAAGGATATCGATATATTTGCTGGAAATTATG
TD3       ATGAGGCACTTTCAAAGAAATTTATAAATAAGAATATCGATATATATGCTGGAAGCTATA
TD110     ATGAGGCACTTTCAAAGAAATTTATAAATAAGAATATCGATATATATGCTGGAAGCTATA
TD7       ATGAGGCACTTTCAAAGAAATTTATAAATAAGAATATCGATATATATGCTGGAAGCTATA
N315      ATGAGGCACTTTCAAAGAAATTTATAAATAAGAATATCGATATATATGCTGGAAGCTATA
TD15      ATGAGGCACTTTCAAAGAAATTTATAAATAAGAATATCGATATATATGCTGGAAGCTATA
TD44      ATGAGGCACTTTCAAAGAAATTTATAAATAAGAATATCGATATATATGCTGGAAGCTATA
TD97      ATGAGGCACTTTCAAAGAAATTTATAAATAAGAATATCGATATATATGCTGGAAGCTATA
TD191     ATGAGGCACTTTCAAAGAAATTTATAAATAAGGATATCGATATATTTGCTGGAAATTATG
TD21      ATGAGGCACTTTCAAAGAAATTTATAAATAAGGATATCGATATATTTGCTGGAAATTATG
TD160     ATGAGGCACTTTCAAAGAAATTTATAAATAAGGATATCGATATATTTGCTGGAAATTATG
TD115     ATGAGGCACTTTCAAAGAAATTTATAAATAAGGATATCGATATATTTGCTGGAAATTATG
TD123     ATGAGGCACTTTCAAAGAAATTTATAAATAAGGATATCGATATATTTGCTGGAAATTATG
TD158     ATGAGGCACTTTCAAAGAAATTTATAAATAAGGATATCGATATATTTGCTGGAAATTATG
TD101     ATGAGGCACTTTCAAATGAATTTATAAATAAGAATATCGATATATATGCTGGAAATTATG
          ****************  ************** ************ ********  *** 

                                                                         420
COL       GATACGGATGTCATGGGGGAGCAACCAATAAAACGCAATGTAGTTATGGTGGTGTTACTT
TD112     GATACGGATGTCATGGGGGAGCAACCAATAAAACGCAATGTAGTTATGGTGGTGTTACTT
TD188     GATACGGATGTCATGGGGGAGCAACCAATAAAACGCAATGTAGTTATGGTGGTGTTACTT
TD8       GATACGGATGTCATGGGGGAGCAACCAATAAAACGCAATGTAGTTATGGTGGTGTTACTT
TD3       GCTATGAATGTCATGGGGGAGCAACCAATAAAACGCAATGTAGTTATGGTGGTGTTACTT
TD110     GCTATGAATGTCATGGGGGAGCAACCAATAAAACGCAATGTAGTTATGGTGGTGTTACTT
TD7       GCTATGAATGTCATGGGGGAGCAACCAATAAAACGCAATGTAGTTATGGTGGTGTTACTT
N315      GCTATGAATGTCATGGGGGAGCAACCAATAAAACGCAATGTAGTTATGGTGGTGTTACTT
TD15      GCTATGAATGTCATGGGGGAGCAACCAATAAAACGCAATGTAGTTATGGTGGTGTTACTT
TD44      GCTATGAATGTCATGGGGGAGCAACCAATAAAACGCAATGTAGTTATGGTGGTGTTACTT
TD97      GCTATGAATGTCATGGGGGAGCAACCAATAAAACGCAATGTAGTTATGGTGGTGTTACTT
TD191     GATATGGATGTCATGGGGGAGCAACCAATAAAACGCAATGTAGTTATGGTGGTGTTACTT
TD21      GATATGGATGTCATGGGGGAGCAACCAATAAAACGCAATGTAGTTATGGTGGTGTTACTT
TD160     GATATGGATGTCATGGGGGAGCAACCAATAAAACGCAATGTAGTTATGGTGGTGTTACTT
TD115     GATATGGATGTCATGGGGGAGCAACCAATAAAACGCAATGTAGTTATGGTGGTGTTACTT
TD123     GATACGGATGTCATGGGGGAGAAACCAATAAAACGCAATGTAGTTATGGAGGGGTTACTT
TD158     GATACGGATGTCATGGGGGAGAAACCAATAAAACGCAATGTAGTTATGGAGGGGTTACTT
TD101     GATACGGATGTCATGGGGGAGCAACCAATAAAACGCAATGTAGTTATGGTGGTGTTACTT
          * ** * ************** *************************** ** *******

                                                                         480
COL       TAAGTGACAATAATAAATACGATGATTATAAGAATATACCTTGTAATTTATGGATTGACG
TD112     TAAGTGACAATAATAAATACGATGATTATAAGAATATACCTTGTAATTTATGGATTGACG
TD188     TAAGTGACAATAATAAATACGATGATTATAAGAATATACCTTGTAATTTATGGATTGACG
TD8       TAAGTGACAATAATAAGTACGATGATTATAAGAATATACCTTGTAATTTATGGATTGACG
TD3       TAAGTGACAATAATAAGTACGATGATTATAAGAATATACCTTGTAATTTATGGATTGACG
TD110     TAAGTGACAATAATAAGTACGATGATTATAAGAATATACCTTGTAATTTATGGATTGACG
TD7       TAAGTGACAATAATAAGTACGATGATTATAAGAATATACCTTGTAATTTATGGATTGACG
N315      TAAGTGACAATAATAAATAAGATGATTATAAGAATATACCTTCTAATTTATGGATTGACG
TD15      TAAGTGACAATAATAAATAAGATGATTATAAGAATATACCTTCTAATTTATGGATTGACG
TD44      TAAGTGACAATAATAAATAAGATGATTATAAGAATATACCTTCTAATTTATGGATTGACG
TD97      TAAGTGACAATAATAAATAAGATGATTATAAGAATATACCTTCTAATTTATGGATTGACG
TD191     TAAGTGACAATAATAAGTACGATGATTATAAGAATATACCTTGTAATTTATGGATTGACG
TD21      TAAGTGACAATAATAAGTACGATGATTATAAGAATATACCTTGTAATTTATGGATTGACG
TD160     TAAGTGACAATAATAAGTACGATGATTATAAGAATATACCTTGTAATTTATGGATTGACG
TD115     TAAGTGACAATAATAAGTACGATGATTATAAGAATATACCTTGTAATTTATGGATTGACG
TD123     TAAGTGACAATAATAAGTACGATAATGATAAGAATATACCTTGTAATTTATGGATTGACG
TD158     TAAGTGACAATAATAAGTACGATAATGATAAGAATATACCTTGTAATTTATGGATTGACG
TD101     TAAGTGACAATAATAAGTACGATGATTATAAGAATATACCTTGTAATTTATGGATTGACG
          **************** ** *** ** *************** *****************
```

```
                                                                         540
COL       GACATCAAACAGAAATAGAACTAACTGCAGTAAAAACGAAAAA-GAAAATTGTTACTATT
TD112     GACATCAAACAGAAATAGAACTAACTGCAGTAAAAACGAAAAA-GAAAATTGTTACTATT
TD188     GACATCAAACAGAAATAGAACTAACTGCAGTAAAAACGAAAAA-GAAAATTGTTACCATT
TD8       GACATCAAGCAGAAATAGAATTAACAGCAGTAAAAACGAAAAA-GAAAATTGTTACCATT
TD3       GACATCAAGCAGAAATAGAATTAACAGCAGTAAAAACGAAAAA-GAAAATTGTTACCATT
TD110     GACATCAAGCAGAAATAGAATTAACAGCAGTAAAAACGAAAAA-GAAAATTGTTACCATT
TD7       GACATCAAGCAGAAATAGAATTAACAGCAGTAAAAACGAAAAA-GAAAATTGTTACCATT
N315      GACATCAAACAGAAATAGAACTAACTGCAGTAAAAACGAAAAAAGAAAATTGTTACCATT
TD15      GACATCAAACAGAAATAGAACTAACTGCAGTAAAAACGAAAAAAGAAAATTGTTACCATT
TD44      GACATCAAACAGAAATAGAACTAACTGCAGTAAAAACGAAAAAAGAAAATTGTTACCATT
TD97      GACATCAAACAGAAATAGAACTAACTGCAGTAAAAACGAAAAAAGAAAATTGTTACCATT
TD191     GACATCAAGCAGAAATAGAATTAACAGCAGTAAAAACGAAAAA-GAAAATTGTTACCATT
TD21      GACATCAAGCAGAAATAGAATTAACAGCAGTAAAAACGAAAAA-GAAAATTGTTACCATT
TD160     GACATCAAGCAGAAATAGAATTAACAGCAGTAAAAACGAAAAA-GAAAATTGTTACCATT
TD115     GACATCAAGCAGAAATAGAATTAACAGCAGTAAAAACGAAAAA-GAAAATTGTTACCATT
TD123     GACATCAAACAGAAATAGAACTAACTGCAGTAAAAACGAAAAA-GAAAATTGTTACCATT
TD158     GACATCAAACAGAAATAGAACTAACTGCAGTAAAAACGAAAAA-GAAAATTGTTACCATT
TD101     GACATCAAACAGAAATAGAACTAACTGCAGTAAAAACGAAAAA-GAAAATTGTTACTATT
          ******** *********** **** ***************** ************ ***

                                                                         600
COL       CAAGAATTAGAGGTTCAATTAAGAAATTATTTGAATGAGAAGTATAAGTTGTACGAACAA
TD112     CAAGAATTAGAGGTTCAATTAAGAAATTATTTGAATGAGAAGTATAAGTTGTACGAACAA
TD188     CAAGAATTAGAGGTTCAATTAAGAAATTATTTGAATGAGAAGTATAAGTTGTACGAACAA
TD8       CAAGAATTAGATGTTCAATTAAGAAATTATTTGAATGAGAAGTATAAGTTGTACGAACAA
TD3       CAAGAATTAGATGTTCAATTAAGAAATTATTTGAATGAGAAGTATAAGTTGTACGAACAA
TD110     CAAGAATTAGATGTTCAATTAAGAAATTATTTGAATGAGAAGTATAAGTTGTACGAACAA
TD7       CAAGAATTAGATGTTCAATTAAGAAATTATTTGAATGAGAAGTATAAGTTGTACGAACAA
N315      CAAGAATTAGATGTTCAATTAAGAAATTATTTGAATGAGAAGTATAAGTTGTACGAACAA
TD15      CAAGAATTAGATGTTCAATTAAGAAATTATTTGAATGAGAAGTATAAGTTGTACGAACAA
TD44      CAAGAATTAGATGTTCAATTAAGAAATTATTTGAATGAGAAGTATAAGTTGTACGAACAA
TD97      CAAGAATTAGATGTTCAATTAAGAAATTATTTGAATGAGAAGTATAAGTTGTACGAACAA
TD191     CAAGAATTAGATGTTCAATTAAGAAATTATTTGAATGAGAAGTATAAGTTGTACGAACAA
TD21      CAAGAATTAGATGTTCAATTAAGAAATTATTTGAATGAGAAGTATAAGTTGTACGAACAA
TD160     CAAGAATTAGATGTTCAATTAAGAAATTATTTGAATGAGAAGTATAAGTTGTACGAACAA
TD115     CAAGAATTAGATGTTCAATTAAGAAATTATTTGAATGAGAAGTATAAGTTGTACGAACAA
TD123     CAAGAATTAGATGTTCAATTAAGAAATTATTTGAATGAGAAGTATAAGTTGTACGAACAA
TD158     CAAGAATTAGATGTTCAATTAAGAAATTATTTGAATGAGAAGTATAAGTTGTACGAACAA
TD101     CAAGAATTAGAGGTTCAATTAAGAAATTATTTGAATGAGAAGTATAAGTTGTACGAACAA
          *********** ************************************************

                                                                         660
COL       GGTGGCGACATTGTTAAAGGGTATGTTAAATATTATAATGATGATGAACAAAATGTAGAA
TD112     GGTGGCGACATTGTTAAAGGGTATGTTAAATATTATAATGATGATGAACAAAATGTAGAA
TD188     GGTGGCGACATTGTTAAAGGGTATGTTAAATATTATAATGATGATGAACAAAATGTAGAA
TD8       GGTGGCGACATTGTTAAAGGGTATGTTAAATATTATAATGATGATGAACAAAATGTAGAA
TD3       GGTGGCGACATTGTTAAAGGGTATGTTAAATATCATAATGATGATGAACAAAATATAGAA
TD110     GGTGGCGACATTGTTAAAGGGTATGTTAAATATCATAATGATGATGAACAAAATATAGAA
TD7       GGTGGCGACATTGTTAAAGGGTATGTTAAATATCATAATGATGATGAACAAAATATAGAA
N315      GGTGGCGACATTGTTAAAGGGTATGTTAAATATCATAATGATGATGAACAAAATGTAGAA
TD15      GGTGGCGACATTGTTAAAGGGTATGTTAAATATCATAATGATGATGAACAAAATGTAGAA
TD44      GGTGGCGACATTGTTAAAGGGTATGTTAAATATCATAATGATGATGAACAAAATGTAGAA
TD97      GGTGGCGACATTGTTAAAGGGTATGTTAAATATCATAATGATGATGAACAAAATGTAGAA
TD191     GGTGGCGACATTGTTAAAGGGTATGTTAAATATCATAATGATGATGAACAAAATATAGAA
TD21      GGTGGCGACATTGTTAAAGGGTATGTTAAATATCATAATGATGATGAACAAAATATAGAA
TD160     GGTGGCGACATTGTTAAAGGGTATGTTAAATATCATAATGATGATGAACAAAATATAGAA
TD115     GGTGGCGACATTGTTAAAGGGTATGTTAAATATCATAATGATGATGAACAAAATATAGAA
TD123     GGTGGCGACATTGTTAAAGGGTATGTTAAATATCATAATGATGA---AAAAAATGTAGAA
TD158     GGTGGCGACATTGTTAAAGGGTATGTTAAATATCATAATGATGATGAAAAAAATGTAGAA
TD101     GGTGGCGACATTGTTAAAGGGTATGTTAAATATTATAATGATGATGAACAAAATGTAGAA
          ********************************* **********   * ***** *****
```

```
                                                                     720
COL      TATGATTTTTATAATTTAAATGGTGAGTATGGTCGTGAGGTATTAAAAATGTATGCTGAT
TD112    TATGATTTTTATAATTTAAATGGTGAGTATGGTCGTGAGGTATTAAAAATGTATGCTGAT
TD188    TATGATTTTTATAATTTAAATGGTGAGTATGGTCGTGAGGTATTAAAAATGTATGCTGAT
TD8      TATGATTTTTATAATTTAAATGGTGAGTATGGTCGTGAGGTATTAAAAATGTATGCTGAT
TD3      TATAATTTTTATAATTTAAATGGTGAGTATGGTTATGAGGTATTAAAAATGTATGCTGAT
TD110    TATAATTTTTATAATTTAAATGGTGAGTATGGTTATGAGGTATTAAAAATGTATGCTGAT
TD7      TATAATTTTTATAATTTAAATGGTGAGTATGGTTATGAGGTATTAAAAATGTATGCTGAT
N315     TATGATTTTTATAATTTAAATGGTGAGTATGGTTATGAGGTATTAAAAATGTATGCTGAT
TD15     TATGATTTTTATAATTTAAATGGTGAGTATGGTTATGAGGTATTAAAAATGTATGCTGAT
TD44     TATGATTTTTATAATTTAAATGGTGAGTATGGTTATGAGGTATTAAAAATGTATGCTGAT
TD97     TATGATTTTTATAATTTAAATGGTGAGTATGGTTATGAGGTATTAAAAATGTATGCTGAT
TD191    TATAATTTTTATAATTTAAATGGTGAGTATGGTTATGAGATATTAAAAATGTATGCTGAT
TD21     TATAATTTTTATAATTTAAATGGTGAGTATGGTTATGAGATATTAAAAATGTATGCTGAT
TD160    TATAATTTTTATAATTTAAATGGTGAGTATGGTTATGAGGTATTAAAAATGTATGCTGAT
TD115    TATAATTTTTATAATTTAAATGGTGAGTATGGTTATGAGGTATTAAAAATGTATGCTGAT
TD123    TATGATTTTTATAATTTAAATGGTGAGTATGGTCATGAGGTATTAAAAATGTATGCTGAT
TD158    TATGATTTTTATAATTTAAATGGTGAGTATGGTCATGAGGTATTAAAAATGTATGCTGAT
TD101    TATGATTTTTATAATTTAAATGGTGAGTATGGTCGTGAGGTATTAAAAATGTATGCTGAT
         *** *****************************  **** ********************

                                                                     780
COL      AATAAAACTATCAATAGTGACAAATTGCATTTAGATATCTATTTATTCAAATCATAATTT
TD112    AATAAAACTATCAATAGTGACAAATTGCATTTAGATATCTATTTATTCAAATCATAATTT
TD188    AATAAAACTATCAATAGTGACAAATTGCATTTAGATATCTATTTATTCAAATCATAATTT
TD8      AATAAAACTATCAATAGTGACAAATTGCATTTAGATATCTATTTATTCAAATCATAATTT
TD3      AATAAAACTATCAATAGTGACAAATTGCATTTAGATATCTATTTATTCAAATCATAATTT
TD110    AATAAAACTATCAATAGTGACAAATTGCATTTAGATATCTATTTATTCAAATCATAATTT
TD7      AATAAAACTATCAATAGTGACAAATTGCATTTAGATATCTATTTATTCAAATCATAATTT
N315     AATAAAACTATCAATAGAGACAAATTGCATTTAGATATCTATTTATTCAAATCATAATTT
TD15     AATAAAACTATCAATAGAGACAAATTGCATTTAGATATCTATTTATTCAAATCATAATTT
TD44     AATAAAACTATCAATAGAGACAAATTGCATTTAGATATCTATTTATTCAAATCATAATTT
TD97     AATAAAACTATCAATAGAGACAAATTGCATTTAGATATCTATTTATTCAAATCATAATTT
TD191    AATAAAACTATCAATAGTGACAAATTGCATTTAGATATCTATTTATTCAAATCATAATTT
TD21     AATAAAACTATCAATAGTGACAAATTGCATTTAGATATCTATTTATTCAAATCATAATTT
TD160    AATAAAACTATCAATAGTGACAAATTGCATTTAGATATCTATTTATTCAAATCATAATTT
TD115    AATAAAACTATCAATAGTGACAAATTGCATTTAGATATCTATTTATTCAAATCATAATTT
TD123    AATAAAACTATCAATAGTGACAAATTGCATTTAGATATCTATTTATTCAAATCATAATTT
TD158    AATAAAACTATCAATAGTGACAAATTGCATTTAGATATCTATTTATTCAAATCATAATTT
TD101    AATAAAACTATCAATAGTGACAAATTGCATTTAGATATCTATTTATTCAAATCATAATTT
         ***************** ******************************************

COL      AATTAAG
TD112    AATTAAG
TD188    AATTAAG
TD8      AATTAAG
TD3      AATTAAG
TD110    AATTAAG
TD7      AATTAAG
N315     AATTAAG
TD15     AATTAAG
TD44     AATTAAG
TD97     AATTAAG
TD191    AATTAAG
TD21     AATTAAG
TD160    AATTAAG
TD115    AATTAAG
TD123    AAGTGAG
TD158    AAGTGAG
TD101    AATTAAG
         ** * **
```

Figure S1. Alignment of nucleotide sequences of intact *selw* genes and pseudogenes encoding truncated SElW. Sequences of strains COL and N315 represent intact and truncated *selw* genes,

respectively. Start codon and stop codon are shown in yellow and blue, respectively. Nucleotide numbers are indicated for intact *selw* represented by strain COL.

(a)

```
                                                                                60
TD123           MGEFEVKYLTGFILILLLEGIFTNSASAIEYSDLHHKSKLDSKRLYNAKVSFANPTDLEN
TD158           MGELEVKYLTGFILILLLEGIFTNSASAIEYSDLHHKSKLDSKRLYNAKVSFANPTDLEN
TD21            ----------------MLEEILTNSASAIEYSDLHHKSKFDSKRLSNAKMSFINPTQLEN
TD191           ----------------MLEEILTNSASAIEYSDLHHKSKFDSKRLSNAKMSFINPTQLEN
TD115           ----------------MLEEILTNSASAIEYSDLHHKSKFDSKRLSNAKMSFINPTQLEN
TD160           ----------------MLEEILTNSASAIEYSDLHHKSKFDSKRLSNAKMSFINPTQLEN
TD3             MGEFEVKYLTGFILILLLEGIFTNSASAIEYSDLHHKSKFDSKRLSNAKMSFINPTQLEN
TD7             MGEFEVKYLTGFILILLLEGIFTNSASAIEYSDLHHKSKFDSKRLSNAKMSFINPTQLEN
TD110           MGEFEVKYLTGFILILLLEGIFTNSASAIEYSDLHHKSKFDSKRLSNAKMSFINPTQLEN
TD112           MGEFEVKYLTGFILILLLEGIFTNSASAIEYSDLHHKSKFDSKRLSNAKMSFINPTQLEN
TD188           MGEFEVKYLTGFILILLLEGIFTNSASAIEYSDLHHKSKFDSKRLSNAKMSFINPTQLEN
COL             MGEFEVKYLTGFILILLLEGIFTNSASAIEYSDLHHKSKFDSKRLSNAKMSFINPTQLEN
TD8             MGEFEVKYLTGFILILLLEGIFTNSASAIEYSDLHHKSKFDSKRLSNAKMSFINPTQLEN
TD101           ----------------MLEEILTNSVSAIEYSDLHHKSKLNSKRLYNAKVSFANPTDLEN
                                :** *:***.*************::**** ***:** ***:***

                                                                               120
TD123           KKTNDRLLKHDLLFHDMFVNDDWKKDFKVEFENEALSKKFINKDIDIFAGNYGYGCHGGE
TD158           KKTNDRLLKHDLLFHDMFVNDDWKKDFKVEFENEALSKKFINKDIDIFAGNYGYGCHGGE
TD21            KNTNDRLLKHDLLFHDMFVNDDWKKDFKVEFENEALSKKFINKDIDIFAGNYGYGCHGGA
TD191           KNTNDRLLKHDLLFHDMFVNDDWKKDFKVEFENEALSKKFINKDIDIFAGNYGYGCHGGA
TD115           KNTNDRLLKHDLLFHDMFVNDDWKKDFKVEFENEALSKKFINKDIDIFAGNYGYGCHGGA
TD160           KNTNDRLLKHDLLFHDMFVNDDWKKDFKVEFENEALSKKFINKDIDIFAGNYGYGCHGGA
TD3             KNTNDRLLKHDLLFHDMFVNDDWKKDFKVEFENEALSKKFINKNIDIYAGSYSYECHGGA
TD7             KNTNDRLLKHDLLFHDMFVNDDWKKDFKVEFENEALSKKFINKNIDIYAGSYSYECHGGA
TD110           KNTNDRLLKHDLLFHDMFVNDDWKKDFKVEFENEALSKKFINKNIDIYAGSYSYECHGGA
TD112           KNTNDRLLKHDLLFHDMFVNDDWKKDFKVEFENEALSKKFINKDIDIFAGNYGYGCHGGA
TD188           KNTNDRLLKHDLLFHDMFVNDDWKKDFKVEFENEALSKKFINKDIDIFAGNYGYGCHGGA
COL             KNTNDRLLKHDLLFHDMFVNDDWKKDFKVEFENEALSKKFINKDIDIFAGNYGYGCHGGA
TD8             KNTKDRLLKHDLLFHDMFVNDDWKKDFKVEFENEALSKKFINKDIDIFAGNYGYGCHGGA
TD101           KNTNDRLLKHDLLFHDMFVNVASKKDFKVEFENEALSNEFINKNIDIYAGNYGYGCHGGA
                *:*:****************   **************::****:***:**.*.* **** 

                                                                               180
TD123           TNKTQCSYGGVTLSDNNKYDNDKNIPCNLWIDGHQTEIELTAVKTKKKIVTIQELDVQLR
TD158           TNKTQCSYGGVTLSDNNKYDNDKNIPCNLWIDGHQTEIELTAVKTKKKIVTIQELDVQLR
TD21            TNKTQCSYGGVTLSDNNKYDDYKNIPCNLWIDGHQAEIELTAVKTKKKIVTIQELDVQLR
TD191           TNKTQCSYGGVTLSDNNKYDDYKNIPCNLWIDGHQAEIELTAVKTKKKIVTIQELDVQLR
TD115           TNKTQCSYGGVTLSDNNKYDDYKNIPCNLWIDGHQAEIELTAVKTKKKIVTIQELDVQLR
TD160           TNKTQCSYGGVTLSDNNKYDDYKNIPCNLWIDGHQAEIELTAVKTKKKIVTIQELDVQLR
TD3             TNKTQCSYGGVTLSDNNKYDDYKNIPCNLWIDGHQAEIELTAVKTKKKIVTIQELDVQLR
TD7             TNKTQCSYGGVTLSDNNKYDDYKNIPCNLWIDGHQAEIELTAVKTKKKIVTIQELDVQLR
TD110           TNKTQCSYGGVTLSDNNKYDDYKNIPCNLWIDGHQAEIELTAVKTKKKIVTIQELDVQLR
TD112           TNKTQCSYGGVTLSDNNKYDDYKNIPCNLWIDGHQTEIELTAVKTKKKIVTIQELEVQLR
TD188           TNKTQCSYGGVTLSDNNKYDDYKNIPCNLWIDGHQTEIELTAVKTKKKIVTIQELEVQLR
COL             TNKTQCSYGGVTLSDNNKYDDYKNIPCNLWIDGHQTEIELTAVKTKKKIVTIQELEVQLR
TD8             TNKTQCSYGGVTLSDNNKYDDYKNIPCNLWIDGHQAEIELTAVKTKKKIVTIQELDVQLR
TD101           TNKTQCSYGGVTLSDNNKYDDYKNIPCNLWIDGHQTEIELTAVKTKKKIVTIQELEVQLR
                ********************: *************:*******************:****
```

```
                                                                           240
TD123           NYLNEKYKLYEQGGDIVKGYVKYHND-EKNVEYDFYNLNGEYGHEVLKMYADNKTINSDK
TD158           NYLNEKYKLYEQGGDIVKGYVKYHNDDEKNVEYDFYNLNGEYGHEVLKMYADNKTINSDK
TD21            NYLNEKYKLYEQGGDIVKGYVKYHNDDEQNIEYNFYNLNGEYGYEILKMYADNKTINSDK
TD191           NYLNEKYKLYEQGGDIVKGYVKYHNDDEQNIEYNFYNLNGEYGYEILKMYADNKTINSDK
TD115           NYLNEKYKLYEQGGDIVKGYVKYHNDDEQNIEYNFYNLNGEYGYEVLKMYADNKTINSDK
TD160           NYLNEKYKLYEQGGDIVKGYVKYHNDDEQNIEYNFYNLNGEYGYEVLKMYADNKTINSDK
TD3             NYLNEKYKLYEQGGDIVKGYVKYHNDDEQNIEYNFYNLNGEYGYEVLKMYADNKTINSDK
TD7             NYLNEKYKLYEQGGDIVKGYVKYHNDDEQNIEYNFYNLNGEYGYEVLKMYADNKTINSDK
TD110           NYLNEKYKLYEQGGDIVKGYVKYHNDDEQNIEYNFYNLNGEYGYEVLKMYADNKTINSDK
TD112           NYLNEKYKLYEQGGDIVKGYVKYYNDDEQNVEYDFYNLNGEYGREVLKMYADNKTINSDK
TD188           NYLNEKYKLYEQGGDIVKGYVKYYNDDEQNVEYDFYNLNGEYGREVLKMYADNKTINSDK
COL             NYLNEKYKLYEQGGDIVKGYVKYYNDDEQNVEYDFYNLNGEYGREVLKMYADNKTINSDK
TD8             NYLNEKYKLYEQGGDIVKGYVKYYNDDEQNVEYDFYNLNGEYGREVLKMYADNKTINSDK
TD101           NYLNEKYKLYEQGGDIVKGYVKYYNDDEQNVEYDFYNLNGEYGREVLKMYADNKTINSDK
                ***********************:** *:*:**:********* *:**************

                       250
TD123           LHLDIYLFKS
TD158           LHLDIYLFKS
TD21            LHLDIYLFKS
TD191           LHLDIYLFKS
TD115           LHLDIYLFKS
TD160           LHLDIYLFKS
TD3             LHLDIYLFKS
TD7             LHLDIYLFKS
TD110           LHLDIYLFKS
TD112           LHLDIYLFKS
TD188           LHLDIYLFKS
COL             LHLDIYLFKS
TD8             LHLDIYLFKS
TD101           LHLDIYLFKS
                **********
```

(b)

```
                                                                      60
TD44          MGEFEVKYLTGFILILLLEGIFTNSASAIEYSDLHHKSKFDSKRLSNAKMSFINPTQLEN
TD97          MGEFEVKYLTGFILILLLEGIFTNSASAIEYSDLHHKSKFDSKRLSNAKMSFINPTQLEN
TD15          MGEFEVKYLTGFILILLLEGIFTNSASAIEYSDLHHKSKFDSKRLSNAKMSFINPTQLEN
N315          MGEFEVKYLTGFILILLLEGIFTNSASAIEYSDLHHKSKFDSKRLSNAKMSFINPTQLEN
COL           MGEFEVKYLTGFILILLLEGIFTNSASAIEYSDLHHKSKFDSKRLSNAKMSFINPTQLEN
              ************************************************************

                                                                     120
TD44          KNTNDRLLKHDLLFHDMFVNVASKKDFKVEFENEALSKKFINKNIDIYAGSYSYECHGGA
TD97          KNTNDRLLKHDLLFHDMFVNVASKKDFKVEFENEALSKKFINKNIDIYAGSYSYECHGGA
TD15          KNTNDRLLKHDLLFHDMFVNVASKKDFKVEFENEALSKKFINKNIDIYAGSYSYECHGGA
N315          KNTNDRLLKHDLLFHDMFVNVASKKDFKVEFENEALSKKFINKNIDIYAGSYSYECHGGA
COL           KNTNDRLLKHDLLFHDMFVNDDWKKDFKVEFENEALSKKFINKDIDIFAGNYGYGCHGGA
              ********************   ********************:***:**.*.* *****

                             138                                     180
TD44          TNKTQCSYGGVTLSDNNK------------------------------------------
TD97          TNKTQCSYGGVTLSDNNK------------------------------------------
TD15          TNKTQCSYGGVTLSDNNK------------------------------------------
N315          TNKTQCSYGGVTLSDNNK------------------------------------------
COL           TNKTQCSYGGVTLSDNNKYDDYKNIPCNLWIDGHQTEIELTAVKTKKKIVTIQELEVQLR
              ******************

                                                                     240
TD44          ------------------------------------------------------------
TD97          ------------------------------------------------------------
TD15          ------------------------------------------------------------
N315          ------------------------------------------------------------
COL           NYLNEKYKLYEQGGDIVKGYVKYYNDDEQNVEYDFYNLNGEYGREVLKMYADNKTINSDK

                     250
TD44          ----------
TD97          ----------
TD15          ----------
N315          ----------
COL           LHLDIYLFKS
```

Figure S2. Alignment of amino acid sequences of intact SElW (**a**), truncated SElW with an intact SElW of strain COL (**b**). Amino acid numbers are indicated for intact SElW.

```
TD188           MGEFEVKYLTGFILILLLEGIFTNSASAIEYS--DLHHKSKFDSKRLSNAKMSFIN--PT
TD112           MGEFEVKYLTGFILILLLEGIFTNSASAIEYS--DLHHKSKFDSKRLSNAKMSFIN--PT
COL             MGEFEVKYLTGFILILLLEGIFTNSASAIEYS--DLHHKSKFDSKRLSNAKMSFIN--PT
TD8             MGEFEVKYLTGFILILLLEGIFTNSASAIEYS--DLHHKSKFDSKRLSNAKMSFIN--PT
TD7             MGEFEVKYLTGFILILLLEGIFTNSASAIEYS--DLHHKSKFDSKRLSNAKMSFIN--PT
TD3             MGEFEVKYLTGFILILLLEGIFTNSASAIEYS--DLHHKSKFDSKRLSNAKMSFIN--PT
TD110           MGEFEVKYLTGFILILLLEGIFTNSASAIEYS--DLHHKSKFDSKRLSNAKMSFIN--PT
TD123           MGEFEVKYLTGFILILLLEGIFTNSASAIEYS--DLHHKSKLDSKRLYNAKVSFAN--PT
TD158           MGELEVKYLTGFILILLLEGIFTNSASAIEYS--DLHHKSKLDSKRLYNAKVSFAN--PT
SEA             MKKTAFTLLLFIALTLTTSPLVNGSEKSEEINEKDLRKKSELQGTALGNLKQIYYYNEKA
                * :  .. *  : * *  . :...* .: * .  **::**:::.. * * *  :     :
                                                                          60

TD188           QLENKNTNDRLLKHDLLFHDMFVNDDWKKDFKVEFENEALSKKFINKDIDIFAGNYGYGC
TD112           QLENKNTNDRLLKHDLLFHDMFVNDDWKKDFKVEFENEALSKKFINKDIDIFAGNYGYGC
COL             QLENKNTNDRLLKHDLLFHDMFVNDDWKKDFKVEFENEALSKKFINKDIDIFAGNYGYGC
TD8             QLENKNTKDRLLKHDLLFHDMFVNDDWKKDFKVEFENEALSKKFINKDIDIFAGNYGYGC
TD7             QLENKNTNDRLLKHDLLFHDMFVNDDWKKDFKVEFENEALSKKFINKNIDIYAGSYSYEC
TD3             QLENKNTNDRLLKHDLLFHDMFVNDDWKKDFKVEFENEALSKKFINKNIDIYAGSYSYEC
TD110           QLENKNTNDRLLKHDLLFHDMFVNDDWKKDFKVEFENEALSKKFINKNIDIYAGSYSYEC
TD123           DLENKKTNDRLLKHDLLFHDMFVNDDWKKDFKVEFENEALSKKFINKDIDIFAGNYGYGC
TD158           DLENKKTNDRLLKHDLLFHDMFVNDDWKKDFKVEFENEALSKKFINKDIDIFAGNYGYGC
SEA             KTENKESHDQFLQHTILFKGFFTDHSWYNDLLVDFDSKDIVDKYKGKKVDLYGAYYGYQC
                . ***:::*::*:* :**:.:*.:..* :*: *:*:.: : .*: .*.:*::.. *.* *
                                                                         120

TD188           HGGATNKTQCSYGGVTLSDNNKYDDYKNIPCNLWIDGHQTEIELTAVKTKKKIVTIQELE
TD112           HGGATNKTQCSYGGVTLSDNNKYDDYKNIPCNLWIDGHQTEIELTAVKTKKKIVTIQELE
COL             HGGATNKTQCSYGGVTLSDNNKYDDYKNIPCNLWIDGHQTEIELTAVKTKKKIVTIQELE
TD8             HGGATNKTQCSYGGVTLSDNNKYDDYKNIPCNLWIDGHQAEIELTAVKTKKKIVTIQELD
TD7             HGGATNKTQCSYGGVTLSDNNKYDDYKNIPCNLWIDGHQAEIELTAVKTKKKIVTIQELD
TD3             HGGATNKTQCSYGGVTLSDNNKYDDYKNIPCNLWIDGHQAEIELTAVKTKKKIVTIQELD
TD110           HGGATNKTQCSYGGVTLSDNNKYDDYKNIPCNLWIDGHQAEIELTAVKTKKKIVTIQELD
TD123           HGGETNKTQCSYGGVTLSDNNKYDNDKNIPCNLWIDGHQTEIELTAVKTKKKIVTIQELD
TD158           HGGETNKTQCSYGGVTLSDNNKYDNDKNIPCNLWIDGHQTEIELTAVKTKKKIVTIQELD
SEA             AGGTPNKTACMYGGVTLHDNNRLTEEKKVPINLWLDGKQNTVPLETVKTNKKNVTVQELD
                 ** .*** * ****** ***:  : *::* ***:**:*  : * :***:** **:***:
                                                                         180

TD188           VQLRNYLNEKYKLYEQ---GGDIVKGYVKYYNDDEQNVEYDFYNLNGEYGREVLKMYADN
TD112           VQLRNYLNEKYKLYEQ---GGDIVKGYVKYYNDDEQNVEYDFYNLNGEYGREVLKMYADN
COL             VQLRNYLNEKYKLYEQ---GGDIVKGYVKYYNDDEQNVEYDFYNLNGEYGREVLKMYADN
TD8             VQLRNYLNEKYKLYEQ---GGDIVKGYVKYYNDDEQNVEYDFYNLNGEYGREVLKMYADN
TD7             VQLRNYLNEKYKLYEQ---GGDIVKGYVKYHNDDEQNIEYNFYNLNGEYGYEVLKMYADN
TD3             VQLRNYLNEKYKLYEQ---GGDIVKGYVKYHNDDEQNIEYNFYNLNGEYGYEVLKMYADN
TD110           VQLRNYLNEKYKLYEQ---GGDIVKGYVKYHNDDEQNIEYNFYNLNGEYGYEVLKMYADN
TD123           VQLRNYLNEKYKLYEQ---GGDIVKGYVKYHND-EKNVEYDFYNLNGEYGHEVLKMYADN
TD158           VQLRNYLNEKYKLYEQ---GGDIVKGYVKYHNDDEKNVEYDFYNLNGEYGHEVLKMYADN
SEA             LQARRYLQEKYNLYNSDVFDGKVQRGLIVFHTSTEPSVNYDLFGAQGQYSNTLLRIYRDN
                :* *.**:***:**:.   .*.: :* : ::.. * .::*:::. :*:*.  :*::* **
                                                                         240

TD188           KTINSDKLHLDIYLFKS
TD112           KTINSDKLHLDIYLFKS
COL             KTINSDKLHLDIYLFKS
TD8             KTINSDKLHLDIYLFKS
TD7             KTINSDKLHLDIYLFKS
TD3             KTINSDKLHLDIYLFKS
TD110           KTINSDKLHLDIYLFKS
TD123           KTINSDKLHLDIYLFKS
TD158           KTINSDKLHLDIYLFKS
SEA             KTINSENMHIDIYLYTS
                *****:::*:****:.*

                              257
```

Figure S3. Alignment of amino acid sequences of intact SElW and SEA. Amino acid numbers are indicated for SEA.

Table S1 Detection of PVL, TSST-1, and enterotoxin (-like) genes in nasal isolates

Toxin genes	*coa* -genotype (no. of isolates)													Total n=110 (%)
	Ia (7)	IIa (3)	IIIa (15)	IVb (11)	Va (10)	Vb (9)	VIa (22)	VIc (1)	VIIa (18)	VIIb (24)	VIIIa (1)	Xa (18)	XIb (5)	
lukS-PV-lukF-PV (PVL)	0	0	2	0	0	0	11	0	1	0	0	0	1	15 (13.6)
tst-1 (TSST-1)	0	2	1	0	0	0	0	0	0	1	0	0	0	4 (3.6)
Enterotoxin (-like) genes														
sea	0	0	0	7	0	0	12	0	1	2	0	0	0	22 (20)
seb	0	1	3	0	1	0	1	0	0	8	0	1	0	15 (13.6)
sec	0	1	0	0	1	2	10	0	0	3	0	6	0	23 (20.9)
seg	3	1	0	0	3	0	3	0	0	7	1	1	0	19 (17.3)
seh	0	0	0	0	0	0	0	0	2	1	0	2	0	5 (4.5)
sei	3	2	0	0	6	0	7	0	1	9	1	3	0	32 (29.1)
selj	0	0	0	0	0	0	0	0	0	0	0	4	0	4 (3.6)
sek	0	0	1	1	0	0	0	0	2	9	0	0	0	13 (11.8)
sel	1	0	1	0	0	3	7	0	1	5	1	6	0	25 (22.7)
sem	3	2	0	0	7	0	4	0	0	8	1	3	0	28 (25.5)
sen	4	1	0	0	6	0	1	0	0	8	0	1	0	21 (19.1)
seo	3	2	0	0	6	0	3	0	0	7	1	1	0	23 (20.9)
sep	0	0	5	0	0	0	0	0	0	3	0	0	0	8 (7.3)
seq	0	0	1	1	0	0	0	0	1	6	0	0	0	9 (8.2)
ser	0	0	0	0	0	0	0	0	0	0	0	3	0	3 (2.7)
ses	0	0	0	0	0	0	0	0	0	0	0	3	0	3 (2.7)
set	0	0	0	0	0	0	0	0	0	0	0	3	0	3 (2.7)
selu	3	1	0	0	3	0	3	0	0	4	1	1	0	16 (14.5)
selw	4	3	10	8	7	7	17	1	13	20	1	14	2	107 (97.3)
selx	4	3	10	8	8	7	17	1	13	19	1	14	1	106 (96.4)
sely	1	0	0	1	0	0	1	0	9	11	0	6	4	33 (30)
Enterotoxin genes clusters														
g, i, m, n, o, u	4	1	1	0	2	0	1	0	0	3	0	1	0	13 (11.8)
g, i, m, n, o	0	0	0	0	0	0	0	0	0	2	0	0	0	2 (1.8)
j, r, s, t	0	0	0	0	0	0	0	0	0	0	0	3	0	3 (2.7)
k, q	0	0	1	1	0	0	0	0	1	6	0	0	0	9 (8.1)

Table S2 Detection of PVL, TSST-1, and enterotoxin (-like) genes in hand isolates

Toxin genes	*coa* -genotype (no. of isolates)													Total n=34 (%)
	Ia (7)	IIa (3)	IIIa (15)	IVb (11)	Va (10)	Vb (9)	VIa (22)	VIc (1)	VIIa (18)	VIIb (24)	VIIIa (1)	Xa (18)	XIb (5)	
lukS-PV-lukF-PV (PVL)	0	0	0	0	1	0	1	0	1	0	0	0	0	3 (11.3)
tst-1 (TSST-1)	0	0	1	0	0	0	0	0	0	0	0	0	0	1 (2.9)
Enterotoxin(-like) genes														
sea	0	0	0	2	1	0	4	0	1	1	0	0	0	9 (26.5)
seb	0	0	2	0	1	0	0	0	0	2	0	0	0	5 (14.7)
sec	0	0	0	0	0	0	3	0	1	1	0	3	0	8 (23.5)
seg	3	0	0	0	2	0	0	0	0	2	0	0	0	7 (20.6)
sei	3	0	0	0	2	0	1	0	0	2	0	1	0	9 (26.5)
selj	0	0	0	0	0	0	0	0	0	0	0	1	0	1 (2.9)
sek	0	0	2	0	1	0	0	0	2	2	0	0	0	7 (20.6)
sel	0	0	0	0	0	1	3	0	1	1	0	3	0	9 (26.5)
sem	3	0	1	0	2	0	0	0	0	2	0	1	0	9 (26.5)
sen	2	0	0	0	2	0	0	0	0	2	0	1	0	7 (20.6)
seo	3	0	0	0	2	0	0	0	0	2	0	0	0	7 (20.6)
sep	0	0	2	0	1	0	0	0	0	2	0	0	0	5 (14.7)
seq	0	0	2	0	1	0	0	0	2	2	0	0	0	7 (20.6)
ser	0	0	0	0	0	0	0	0	0	0	0	1	0	1 (2.9)
ses	0	0	0	0	0	0	0	0	0	0	0	1	0	1 (2.9)
set	0	0	0	0	0	0	0	0	0	0	0	1	0	1 (2.9)
selu	3	0	0	0	2	0	0	0	0	2	0	0	0	7 (20.6)
selw	3	0	5	3	3	2	5	0	5	4	0	4	0	34 (100)
selx	3	0	5	3	2	2	5	0	5	4	0	4	0	33 (97.9)
sely	1	0	1	0	0	0	0	0	2	0	0	3	0	7 (20.6)
Enterotoxin genes clusters														
g, i, m, n, o, u	2	0	0	0	2	0	0	0	0	2	0	0	0	6 (17.6)
g, i, m, n, o	0	0	0	0	0	0	0	0	0	1	0	0	0	1 (2.9)
j, r, s, t	0	0	0	0	0	0	0	0	0	0	0	1	0	1 (2.9)
k, q	0	0	2	0	1	0	0	0	2	2	0	0	0	7 (20.6)

Table S3 Identical *coa* types and toxin gene profile in same individual

No.	Age/ Sex	Isolate ID	Hand swab (H) / Nasal swab (N)	*coa* type	enterotoxin(-like) genes, PVL genes
1	22/M	TD10	H	Xa	*sec, sei, sel, selx, sely, selw*
		TD9	N		
2	27/M	TD16	H	IVb	*sea, selx*
		TD15	N		
3	22/M	TD20	H	VIa	*sea, sei, sel, selx, selw*
		TD19	N		
4	25/F	TD22	H	Xa	*sei, ses, set, ser, selx, selw*
		TD21	N		
5	26/M	TD35	H	IVb	*sea, selx, sew*
		TD34	N		
6	25/M	TD42	H	VIIa	*selx, selw*
		TD41	N		
7	22/M	TD46	H	IIIa	*seb, sep, selx*
		TD45	N		
8	40/M	TD155	H	Vb	*selx, selw*
		TD107	N		
9	22/F	TD156	H	VIIb	*sea, seg, sei, sem, sen, seo, seu, selx*
		TD104	N		
10	28/M	TD157	H	VIa	*sea, sel, selx, selw, pvl* (*lukS-PV-lukF-PV*)
		TD110	N		
11	22/M	TD156	H	Ia	*seg, sei, sem, sen, seo, seu, selx, selw*
		TD104	N		
12	25/M	TD165	H	Vb	*sel, selx, selw*
		TD164	N		
13	25/F	TD100	H	VIa	*selx, selw*
		TD99	N		
14	28/M	TD166	H	VIIa	*selx, selw*
		TD132	N		
15	29/M	TD104	H	VIIb	*seb, sek, sep, seq, selx, sely, selw*
		TD103	N		
16	32/F	TD177	H	VIa	*sea, selx, selw*
		TD106	N		
17	40/M	TD102	H	Xa	*sec, sel, selx, sely*
		TD141	N		

Table S4 Different *coa* types and/or toxin gene profile in same individual

No.	Age/Sex	Strain ID	Hand swab (H) / Nasal swab (N)	*coa* type	enterotoxin (-like) genes, TSST-1 gene
1	24/F	TD18	H	IIIa	*selx, selw*
		TD17	N	VIIb	*sec, seg, sei, sel, sem, sen, seo, selx*
2	42/M	TD30	H	VIIb	*sec, seg, sei, sel, sem, sen, seo, seu, selx*
		TD29	N	VIIb	*sec, seg, sei, sel, sem, sen, seo, selx*
3	45/F	TD158	H	Va	*sea, seg, sei, sem, sen, seo, seu, selx, selw*
		TD111	N	Va	*seg, sei, sem, sen, seo, seu, selx*
4	23/F	TD159	H	IIIa	*sek, sel, seq, selx, tst-1*
		TD112	N	IIIa	*sek, sem, seq, selx, tst-1*
5	24/F	TD160	H	VIIa	*sek, seq, selx, selw*
		TD115	N	VIIa	*seh, sek, selx, selw*
6	27/M	TD97	H	Ia	*seg, sei, sem, sen, seo, seu, selx, sely, selw*
		TD118	N	Xa	*seg, sei, sem, sen, seo, seu, selx, selw*
7	25/M	TD98	H	VIIa	*sea, sec, sek, sel, seq, selx, selw*
		TD118	N	VIIa	*sea, seh, sei, sek, sel, seq, selx, selw*
8	23/M	TD161	H	VIa	*sea, sel, selx, selw*
		TD121	N	VIIb	*sea, sei, sel, selx, selw*
9	21/F	TD163	H	VIIb	*seb, sek, sep, seq, selx, sely, selw*
		TD127	N	VIIb	*seb, sek, sep, selx, sely, selw*
10	28/F	TD128a	H	VIa	*seb, seg, sei, sem, seo, seu*
		TD128b	N	VIIa	*selx, selw*
11	26/F	TD134	H	Ia	*seg, sei, sem, seo, seu, selx, selw*
		TD133	N	Ia	*seg, sei, sem, sen, seo, seu, selx, selw*
12	29/M	TD167	H	IVb	*selx, selw*
		TD136	N	Xa	*sej, ses, set, ser, selx, selw*
13	29/F	TD139	H	IIIa	*sek, sep, seq, selx, sely, selw*
		TD138	N	VIIb	*sek, selx, sely, selw*
14	25/F	TD173	H	Va	*seb, sek, sep, seq*
		TD144	N	Va	*sei, sem, seo, seu, selx, selw*
15	27/F	TD174	H	Va	*seg, sei, sem, sen, seo, seu, selx*
		TD145	N	VIIb	*seg, sei, sem, sen, seo, seu, selx*
16	22/M	TD105	H	Xa	*sec, sel, sem, sen, selx, sely*
		TD146	N	Xa	*sec, sel, sem, selx, sely*
17	27/M	TD176	H	IIIa	*seb*
		TD147	N	IIIa	*seb, sep, selx*

Table S5 Identities (Percentage) of *selw* nucleotide sequences (upper right) and deduced amino acid sequences (lower left) among the selected strains

	Identity with strain																					
	Group1					Group2			Group3					Group4				Group5			Group6	SEA
Strain	TD101	TD123	TD158	H-EMRSA-15	RF-122	TD112	TD188	COL	TD160	TD115	TD191	TD21	MW2	TD44	TD97	TD15	N315	TD7	TD3	TD110	TD8	
TD101		91.0	91.5	90.2	87.4	96.2	96.0	92.7	91.5	91.5	91.1	91.1	91.5	87.7	87.7	87.7	87.7	89.7	89.7	89.7	91.5	58.2
TD123	96.0		99.2	95.2	93.3	94.4	94.4	94.4	92.3	92.3	91.1	91.1	91.5	89.3	89.3	89.3	88.5	92.1	92.1	92.1	94.0	57.5
TD158	96.3	99.5		95.2	92.4	94.4	94.4	94.4	92.7	92.7	92.3	92.3	91.1	88.6	88.6	88.6	87.8	92.1	92.1	92.1	94.0	57.4
H-MRSA-15	95.8	97.3	97.6		90.8	92.9	92.9	93.6	91.5	91.5	91.0	91.0	90.7	87.1	87.1	87.1	88.4	90.5	90.5	90.5	92.5	57.8
RF122	96.3	97.1	97.2	97.1		89.1	89.1	87.6	84.5	84.5	84.5	84.5	82.9	87.4	87.4	87.4	87.4	89.9	89.9	89.9	88.2	58.5
TD112	96.2	96.2	96.2	96.2	95.8		100	100	96.2	96.2	95.7	95.7	95.3	94.3	94.3	94.3	93.5	95.6	95.6	95.6	98.8	58
TD188	96.0	96.3	96.3	96.3	95.9	99.9		100	96.2	96.2	95.7	95.7	95.3	94.3	94.3	94.3	93.5	95.6	95.6	95.6	98.8	57.8
COL	96.3	96.1	96.3	96.3	95.8	100	99.9		96.2	96.2	95.7	95.7	95.3	94.3	94.3	94.3	93.5	95.6	95.6	95.6	98.8	56.1
TD160	95.6	95.6	95.9	95.9	95.6	97.3	97.5	97.5		100	99.6	99.6	100	91.0	91.0	91.0	91.0	96.6	96.6	96.6	96.6	58
TD115	95.6	95.6	95.9	95.9	95.6	97.3	97.5	97.5	100		99.6	99.6	100	91.0	91.0	91.0	91.0	96.6	96.6	96.6	96.6	58
TD191	95.5	95.5	95.8	95.8	95.5	97.2	97.3	97.3	99.9	99.9		100	99.6	91.0	91.0	91.0	91.0	96.2	96.2	96.2	96.2	58
TD21	95.5	95.5	95.8	95.8	95.5	97.2	97.3	97.3	99.9	99.9	100		99.6	91.0	91.0	91.0	91.0	96.2	96.2	96.2	96.2	58
MW2	95.5	95.8	95.8	95.8	95.6	97.5	97.6	97.5	99.9	99.9	99.7	99.7		89.5	89.5	89.5	89.5	95.8	95.8	95.8	95.8	56
TD44	95.0	94.7	95.2	95.2	95.4	97.2	97.4	97.2	96.4	96.4	96.3	96.3	96.6		100	100	99.3	97.9	97.9	97.9	93.6	57.9
TD97	95.0	94.7	95.2	95.2	95.4	97.2	97.4	97.2	96.4	96.4	96.3	96.3	96.6	100		100	99.3	97.9	97.9	97.9	93.6	57.9
TD15	95.0	94.7	95.0	95.2	95.2	97.2	97.4	97.2	96.4	96.4	96.3	96.3	96.6	100	100		99.3	97.9	97.9	97.9	93.6	57.9
N315	95.1	94.7	95.4	95.4	95.4	97.2	97.3	97.2	96.6	96.6	96.4	96.4	96.6	100	100	100		97.1	97.1	97.1	92.8	57.9
TD7	94.6	95.1	95.1	95.1	95.8	97.4	97.5	97.3	98.1	98.1	98.0	98.0	98.3	98.3	98.3	98.3	98.3		100	100	96.0	57.6
TD3	94.6	95.1	95.1	95.1	95.8	97.4	97.5	97.3	98.1	98.1	98.0	98.0	98.3	98.3	98.3	98.3	98.3	100		100	96.0	57.6
TD110	94.6	95.1	95.1	95.1	95.8	97.4	97.5	97.3	98.1	98.1	98.0	98.0	98.3	98.3	98.3	98.3	98.3	100	100		96.0	57.6
TD8	95.5	96.0	95.8	95.8	95.6	99.1	99.2	99.1	98.0	98.0	97.9	97.9	98.1	96.8	96.8	96.8	96.8	98.0	98.0	98.0		57.4
SEA (similarity)	76.2	73.2	73.2	67.5	70.8	74	74	77.7	77.2	76.4	77.2	76.4	76.4	78.2	78.2	71.2	71.2	74.4	78.2	78.2	73.6	
SEA (identity))	41.4	40.6	40.9	40.9	31.7	39.4	39.4	41.7	42.6	40.3	42.6	40.3	40.3	39.1	39.1	34.5	34.5	39.4	39.1	39.1	39.8	

Table S6 Primers used for PCR amplification of *S. argenteus* genes			
Target gene	Primer	sequence (5'-3')	Product size
thermonuclease	Sta-arg-nucF1	CTATTAAATAATTGTAAAAGTGTAG	
	Sta-arg-nucR1	CTGATTCGATAATAAAACATAACGTG	840bp (+Sta-arg-nucF1)
orfX-cas1	orfX-AB	ATGAAAATCACCATTTTAGCTGTAGG	
	Sta-argR3	ATTCTCCGCCACAATAGCTGC	ca. 600bp (+orfX-AB)
	Sta-argR1	AAGCCTTTGCGCTTTGTGCGACTGA	ca. 1kb (+orfX-AB)
	Sta-argF1	CATAAATCCAAAAACAGGCTCTACATC	
	Sta-argR2	CCAAATTCTATCTCTAGTTCTCCCAG	ca. 4kb (+Sta-argF1)
	Sta-argR4	GATGGTAAAGGCGGCGTCATTTGG	ca. 1.7kb (+Sta-argF1)
	Sta-argF2	CTTAATAATCCTTTACCAGAGCGACC	ca. 2.5kb (+Sta-argR2)
	Sta-argF5	TGAGAGTTCTAAATCTCCAC	
	Sta-argR5	CCGAGTTCTAATAATCGAGTACGA	ca. 2.6kb (+Sta-argF5)
